# Score-based prediction of genomic islands in prokaryotic genomes using hidden Markov models

**DOI:** 10.1186/1471-2105-7-142

**Published:** 2006-03-16

**Authors:** Stephan Waack, Oliver Keller, Roman Asper, Thomas Brodag, Carsten Damm, Wolfgang Florian Fricke, Katharina Surovcik, Peter Meinicke, Rainer Merkl

**Affiliations:** 1 Institut für Informatik, Universität Göttingen, Lotzestr. 16–18, 37083 Göttingen, Germany; 2 Institut für Numerische und Angewandte Mathematik, Universität Göttingen, Lotzestr. 16–18, 37083 Göttingen, Germany; 3 Göttingen Genomics Laboratory, Universität Göttingen, Grisebachstr. 8, 37077 Göttingen, Germany; 4 Institut für Mikrobiologie und Genetik, Universität Göttingen, Goldschmidtstr. 1, 37077 Göttingen, Germany; 5 Institut für Biophysik und Physikalische Biochemie, Universität Regensburg, Universitätsstr. 31, 93053 Regensburg, Germany

## Abstract

**Background:**

Horizontal gene transfer (HGT) is considered a strong evolutionary force shaping the content of microbial genomes in a substantial manner. It is the difference in speed enabling the rapid adaptation to changing environmental demands that distinguishes HGT from gene genesis, duplications or mutations. For a precise characterization, algorithms are needed that identify transfer events with high reliability. Frequently, the transferred pieces of DNA have a considerable length, comprise several genes and are called genomic islands (GIs) or more specifically pathogenicity or symbiotic islands.

**Results:**

We have implemented the program SIGI-HMM that predicts GIs and the putative donor of each individual alien gene. It is based on the analysis of codon usage (CU) of each individual gene of a genome under study. CU of each gene is compared against a carefully selected set of CU tables representing microbial donors or highly expressed genes. Multiple tests are used to identify putatively alien genes, to predict putative donors and to mask putatively highly expressed genes. Thus, we determine the states and emission probabilities of an inhomogeneous hidden Markov model working on gene level. For the transition probabilities, we draw upon classical test theory with the intention of integrating a sensitivity controller in a consistent manner. SIGI-HMM was written in JAVA and is publicly available. It accepts as input any file created according to the EMBL-format.

It generates output in the common GFF format readable for genome browsers. Benchmark tests showed that the output of SIGI-HMM is in agreement with known findings. Its predictions were both consistent with annotated GIs and with predictions generated by different methods.

**Conclusion:**

SIGI-HMM is a sensitive tool for the identification of GIs in microbial genomes. It allows to interactively analyze genomes in detail and to generate or to test hypotheses about the origin of acquired genes.

## Background

Horizontal gene transfer (HGT) is a process that results in the acquisition of novel genes originating from perhaps taxonomically unrelated species. This phenomenon is frequent among microbes and is considered a means of rapid adaptation to changing environmental demands [1]. Pieces of DNA acquired *via *HGT frequently have a considerable length. These patches have been called genomic islands (GI) or due to their role and more specifically pathogenicity islands [2] or symbiotic islands [3].

Several methods have been developed for the prediction of GIs based on different approaches to identify putatively alien (pA) genes [4-12]. Each of these concepts has specific preferences and drawbacks; for recent reviews see [13,14]. In the following, we describe an approach which relies on the genome theory postulating a rather homogeneous codon usage within a genome [15]. The algorithm exploits taxon specific differences in codon usage for the identification of pA genes and the prediction of their putative origin. Hidden Markov models (HMMs) are a state of the art concept in computational learning theory. A sequence of observations is considered as being emitted from the states of an invisible Markov chain. The Viterbi algorithm efficiently computes a sequence of states that have the maximal posteriori probability given a certain sequence of observations and fixed transition and emission probabilities. The challenge in designing a HMM is representing the real situation adequately in order to generate relevant predictions. HMM have proved useful in many applications. In the case of predicting eukaryotic genes, for example, the programs GENSCAN [16,17], HMMGene [18,19], GenomeScan [20], AUGUSTUS [21], and AUGUSTUS+ [22] are HMM-based.

It has been shown that HMMs allow to predict GIs [9]. GIs have typically a considerable length, therefore we have decided to implement a HMM assessing GI prediction on the *gene *level. GIs can originate from a variety of *a priori *unknown donors. Therefore, it is difficult to assure sufficient test statistics. We will describe an approach named SIGI-HMM. To some extent, it is based on principles introduced with SIGI [23]. This program was used to analyze individual genomes [24,25] and to study the content of genomic islands in general [26] as well as to characterize gene-flux between bacteria and archaea [27]. For SIGI-HMM we substituted a heuristic approach with a HMM. SIGI-HMM has only few parameters to adjust. The most relevant one is a sensitivity controller which affects transitions of the HMM in a consistent manner. We will demonstrate and assess the performance of SIGI-HMM by analyzing genomes in detail.

## Implementation

We have implemented SIGI-HMM in Java as a first module of our software suite COLOMBO intended as a workbench for the statistical analysis of genomic data. The program can be downloaded from [28]. The download package contains also the program Artemis [29], which is used to visualize the output of SIGI-HMM. After the installation, a genomic dataset formatted in EMBL-format can be loaded and analyzed. SIGI-HMM creates several lists containing the predictions in GFF-format or tabulated. Predictions are classified according to the categories NATIVE and PUTAL. In addition, a modified EMBL-formatted file is generated containing both the original annotation and the predictions. This file can be fed into Artemis in order to color-code and visualize genome content. Thus, the user can interactively study the composition of genomes. Intentionally, only few parameters can be manipulated by the user: The sensitivity controller and the gap length which decides on merging single GIs to larger ones. In addition, the user can supplement the list of putative donors we have deduced from the CUTG database (see below). The default value of the the sensitivity controller was chosen to give predictions consistent with published results; see Table 1. If it is known that the genome under study contains GIs, we propose the following approach in order to optimize sensitivity of SIGI-HMM: Starting from a low value, sensitivity should be increased until all known GIs appear. If new islands emerge, they show the same degree of codon usage bias and should be considered GIs.

## Results

The following text is organized as follows: First we introduce data models, the scoring system and the architecture of the HMM. Then we evaluate the predictive power of the algorithm and present analyses of several genomes.

### Stochastic data models

Let G
 MathType@MTEF@5@5@+=feaafiart1ev1aaatCvAUfKttLearuWrP9MDH5MBPbIqV92AaeXatLxBI9gBamrtHrhAL1wy0L2yHvtyaeHbnfgDOvwBHrxAJfwnaebbnrfifHhDYfgasaacH8akY=wiFfYdH8Gipec8Eeeu0xXdbba9frFj0=OqFfea0dXdd9vqai=hGuQ8kuc9pgc9s8qqaq=dirpe0xb9q8qiLsFr0=vr0=vr0dc8meaabaqaciaacaGaaeqabaWaaeGaeaaakeaaimaacqWFge=raaa@382D@ be a series of genes as deduced from a genome coding for proteins  . For each codon *c *we count its occurrence *#c *in G
 MathType@MTEF@5@5@+=feaafiart1ev1aaatCvAUfKttLearuWrP9MDH5MBPbIqV92AaeXatLxBI9gBamrtHrhAL1wy0L2yHvtyaeHbnfgDOvwBHrxAJfwnaebbnrfifHhDYfgasaacH8akY=wiFfYdH8Gipec8Eeeu0xXdbba9frFj0=OqFfea0dXdd9vqai=hGuQ8kuc9pgc9s8qqaq=dirpe0xb9q8qiLsFr0=vr0=vr0dc8meaabaqaciaacaGaaeqabaWaaeGaeaaakeaaimaacqWFge=raaa@382D@. We define the *synonymous frequency q*_*ac *_∈ [0,1] as the ratio of *#c *divided by the occurrence of the amino acid *a *encoded by *c *in  . The *frequency q*_*c *_∈ [0,1] of *c *in G
 MathType@MTEF@5@5@+=feaafiart1ev1aaatCvAUfKttLearuWrP9MDH5MBPbIqV92AaeXatLxBI9gBamrtHrhAL1wy0L2yHvtyaeHbnfgDOvwBHrxAJfwnaebbnrfifHhDYfgasaacH8akY=wiFfYdH8Gipec8Eeeu0xXdbba9frFj0=OqFfea0dXdd9vqai=hGuQ8kuc9pgc9s8qqaq=dirpe0xb9q8qiLsFr0=vr0=vr0dc8meaabaqaciaacaGaaeqabaWaaeGaeaaakeaaimaacqWFge=raaa@382D@ is defined as *#c *divided by the occurrence of all codons in  .

Now let G
 MathType@MTEF@5@5@+=feaafiart1ev1aaatCvAUfKttLearuWrP9MDH5MBPbIqV92AaeXatLxBI9gBamrtHrhAL1wy0L2yHvtyaeHbnfgDOvwBHrxAJfwnaebbnrfifHhDYfgasaacH8akY=wiFfYdH8Gipec8Eeeu0xXdbba9frFj0=OqFfea0dXdd9vqai=hGuQ8kuc9pgc9s8qqaq=dirpe0xb9q8qiLsFr0=vr0=vr0dc8meaabaqaciaacaGaaeqabaWaaeGaeaaakeaaimaacqWFge=raaa@382D@_0 _be a prokaryotic genome whose genomic islands have to be predicted and let G
 MathType@MTEF@5@5@+=feaafiart1ev1aaatCvAUfKttLearuWrP9MDH5MBPbIqV92AaeXatLxBI9gBamrtHrhAL1wy0L2yHvtyaeHbnfgDOvwBHrxAJfwnaebbnrfifHhDYfgasaacH8akY=wiFfYdH8Gipec8Eeeu0xXdbba9frFj0=OqFfea0dXdd9vqai=hGuQ8kuc9pgc9s8qqaq=dirpe0xb9q8qiLsFr0=vr0=vr0dc8meaabaqaciaacaGaaeqabaWaaeGaeaaakeaaimaacqWFge=raaa@382D@_1_, G
 MathType@MTEF@5@5@+=feaafiart1ev1aaatCvAUfKttLearuWrP9MDH5MBPbIqV92AaeXatLxBI9gBamrtHrhAL1wy0L2yHvtyaeHbnfgDOvwBHrxAJfwnaebbnrfifHhDYfgasaacH8akY=wiFfYdH8Gipec8Eeeu0xXdbba9frFj0=OqFfea0dXdd9vqai=hGuQ8kuc9pgc9s8qqaq=dirpe0xb9q8qiLsFr0=vr0=vr0dc8meaabaqaciaacaGaaeqabaWaaeGaeaaakeaaimaacqWFge=raaa@382D@_2_, ..., G
 MathType@MTEF@5@5@+=feaafiart1ev1aaatCvAUfKttLearuWrP9MDH5MBPbIqV92AaeXatLxBI9gBamrtHrhAL1wy0L2yHvtyaeHbnfgDOvwBHrxAJfwnaebbnrfifHhDYfgasaacH8akY=wiFfYdH8Gipec8Eeeu0xXdbba9frFj0=OqFfea0dXdd9vqai=hGuQ8kuc9pgc9s8qqaq=dirpe0xb9q8qiLsFr0=vr0=vr0dc8meaabaqaciaacaGaaeqabaWaaeGaeaaakeaaimaacqWFge=raaa@382D@_*r *_be genomes assumed to be the donors for pA genes occurring in G
 MathType@MTEF@5@5@+=feaafiart1ev1aaatCvAUfKttLearuWrP9MDH5MBPbIqV92AaeXatLxBI9gBamrtHrhAL1wy0L2yHvtyaeHbnfgDOvwBHrxAJfwnaebbnrfifHhDYfgasaacH8akY=wiFfYdH8Gipec8Eeeu0xXdbba9frFj0=OqFfea0dXdd9vqai=hGuQ8kuc9pgc9s8qqaq=dirpe0xb9q8qiLsFr0=vr0=vr0dc8meaabaqaciaacaGaaeqabaWaaeGaeaaakeaaimaacqWFge=raaa@382D@_0_. We consider G
 MathType@MTEF@5@5@+=feaafiart1ev1aaatCvAUfKttLearuWrP9MDH5MBPbIqV92AaeXatLxBI9gBamrtHrhAL1wy0L2yHvtyaeHbnfgDOvwBHrxAJfwnaebbnrfifHhDYfgasaacH8akY=wiFfYdH8Gipec8Eeeu0xXdbba9frFj0=OqFfea0dXdd9vqai=hGuQ8kuc9pgc9s8qqaq=dirpe0xb9q8qiLsFr0=vr0=vr0dc8meaabaqaciaacaGaaeqabaWaaeGaeaaakeaaimaacqWFge=raaa@382D@_1 _to G
 MathType@MTEF@5@5@+=feaafiart1ev1aaatCvAUfKttLearuWrP9MDH5MBPbIqV92AaeXatLxBI9gBamrtHrhAL1wy0L2yHvtyaeHbnfgDOvwBHrxAJfwnaebbnrfifHhDYfgasaacH8akY=wiFfYdH8Gipec8Eeeu0xXdbba9frFj0=OqFfea0dXdd9vqai=hGuQ8kuc9pgc9s8qqaq=dirpe0xb9q8qiLsFr0=vr0=vr0dc8meaabaqaciaacaGaaeqabaWaaeGaeaaakeaaimaacqWFge=raaa@382D@_*r *_as representatives of taxa T
 MathType@MTEF@5@5@+=feaafiart1ev1aaatCvAUfKttLearuWrP9MDH5MBPbIqV92AaeXatLxBI9gBamrtHrhAL1wy0L2yHvtyaeHbnfgDOvwBHrxAJfwnaebbnrfifHhDYfgasaacH8akY=wiFfYdH8Gipec8Eeeu0xXdbba9frFj0=OqFfea0dXdd9vqai=hGuQ8kuc9pgc9s8qqaq=dirpe0xb9q8qiLsFr0=vr0=vr0dc8meaabaqaciaacaGaaeqabaWaaeGaeaaakeaaimaacqWFtepvaaa@3847@_1 _to T
 MathType@MTEF@5@5@+=feaafiart1ev1aaatCvAUfKttLearuWrP9MDH5MBPbIqV92AaeXatLxBI9gBamrtHrhAL1wy0L2yHvtyaeHbnfgDOvwBHrxAJfwnaebbnrfifHhDYfgasaacH8akY=wiFfYdH8Gipec8Eeeu0xXdbba9frFj0=OqFfea0dXdd9vqai=hGuQ8kuc9pgc9s8qqaq=dirpe0xb9q8qiLsFr0=vr0=vr0dc8meaabaqaciaacaGaaeqabaWaaeGaeaaakeaaimaacqWFtepvaaa@3847@_*r *_which are assumed to be the putative sources of G
 MathType@MTEF@5@5@+=feaafiart1ev1aaatCvAUfKttLearuWrP9MDH5MBPbIqV92AaeXatLxBI9gBamrtHrhAL1wy0L2yHvtyaeHbnfgDOvwBHrxAJfwnaebbnrfifHhDYfgasaacH8akY=wiFfYdH8Gipec8Eeeu0xXdbba9frFj0=OqFfea0dXdd9vqai=hGuQ8kuc9pgc9s8qqaq=dirpe0xb9q8qiLsFr0=vr0=vr0dc8meaabaqaciaacaGaaeqabaWaaeGaeaaakeaaimaacqWFge=raaa@382D@_0_'s alien genes. For each protein (i.e. sequence of amino acids) *π *= *a*_1_, *a*_2_,..., *a*_*n *_that is encoded by a gene *g *of genome G
 MathType@MTEF@5@5@+=feaafiart1ev1aaatCvAUfKttLearuWrP9MDH5MBPbIqV92AaeXatLxBI9gBamrtHrhAL1wy0L2yHvtyaeHbnfgDOvwBHrxAJfwnaebbnrfifHhDYfgasaacH8akY=wiFfYdH8Gipec8Eeeu0xXdbba9frFj0=OqFfea0dXdd9vqai=hGuQ8kuc9pgc9s8qqaq=dirpe0xb9q8qiLsFr0=vr0=vr0dc8meaabaqaciaacaGaaeqabaWaaeGaeaaakeaaimaacqWFge=raaa@382D@_0 _(given by the sequence of codons *c*_1_,*c*_2_,...,*c*_*n*_), and for each *ρ *= 0, 1, ..., *r*, we define the probability

Pρ(g|π):=qa1c1(ρ)⋅qa2c2(ρ)⋅...⋅qancn(ρ),     (1)
 MathType@MTEF@5@5@+=feaafiart1ev1aaatCvAUfKttLearuWrP9MDH5MBPbIqV92AaeXatLxBI9gBaebbnrfifHhDYfgasaacH8akY=wiFfYdH8Gipec8Eeeu0xXdbba9frFj0=OqFfea0dXdd9vqai=hGuQ8kuc9pgc9s8qqaq=dirpe0xb9q8qiLsFr0=vr0=vr0dc8meaabaqaciaacaGaaeqabaqabeGadaaakeaacqWGqbaudaWgaaWcbaacciGae8xWdihabeaakiabcIcaOiabdEgaNjabcYha8jab=b8aWjabcMcaPiabcQda6iabg2da9iabdghaXnaaDaaaleaacqWGHbqydaWgaaadbaGaeGymaedabeaaliabdogaJnaaBaaameaacqaIXaqmaeqaaaWcbaGaeiikaGIae8xWdiNaeiykaKcaaOGaeyyXICTaemyCae3aa0baaSqaaiabdggaHnaaBaaameaacqaIYaGmaeqaaSGaem4yam2aaSbaaWqaaiabikdaYaqabaaaleaacqGGOaakcqWFbpGCcqGGPaqkaaGccqGHflY1cqGGUaGlcqGGUaGlcqGGUaGlcqGHflY1cqWGXbqCdaqhaaWcbaGaemyyae2aaSbaaWqaaiabd6gaUbqabaWccqWGJbWydaWgaaadbaGaemOBa4gabeaaaSqaaiabcIcaOiab=f8aYjabcMcaPaaakiabcYcaSiaaxMaacaWLjaWaaeWaaeaacqaIXaqmaiaawIcacaGLPaaaaaa@6515@

where qac(ρ)
 MathType@MTEF@5@5@+=feaafiart1ev1aaatCvAUfKttLearuWrP9MDH5MBPbIqV92AaeXatLxBI9gBaebbnrfifHhDYfgasaacH8akY=wiFfYdH8Gipec8Eeeu0xXdbba9frFj0=OqFfea0dXdd9vqai=hGuQ8kuc9pgc9s8qqaq=dirpe0xb9q8qiLsFr0=vr0=vr0dc8meaabaqaciaacaGaaeqabaqabeGadaaakeaacqWGXbqCdaqhaaWcbaGaemyyaeMaem4yamgabaGaeiikaGccciGae8xWdiNaeiykaKcaaaaa@3457@ ∈ [0,1] is the synonymous frequency in genome G
 MathType@MTEF@5@5@+=feaafiart1ev1aaatCvAUfKttLearuWrP9MDH5MBPbIqV92AaeXatLxBI9gBamrtHrhAL1wy0L2yHvtyaeHbnfgDOvwBHrxAJfwnaebbnrfifHhDYfgasaacH8akY=wiFfYdH8Gipec8Eeeu0xXdbba9frFj0=OqFfea0dXdd9vqai=hGuQ8kuc9pgc9s8qqaq=dirpe0xb9q8qiLsFr0=vr0=vr0dc8meaabaqaciaacaGaaeqabaWaaeGaeaaakeaaimaacqWFge=raaa@382D@_*ρ *_as defined above.

### Scoring scheme

We utilize the odds ratio

P0(g|π)Pρ(g|π)=qa1c1(0)⋅qa2c2(0)⋅...⋅qancn(0)qa1c1(ρ)⋅qa2c2(ρ)⋅...⋅qancn(ρ)
 MathType@MTEF@5@5@+=feaafiart1ev1aaatCvAUfKttLearuWrP9MDH5MBPbIqV92AaeXatLxBI9gBaebbnrfifHhDYfgasaacH8akY=wiFfYdH8Gipec8Eeeu0xXdbba9frFj0=OqFfea0dXdd9vqai=hGuQ8kuc9pgc9s8qqaq=dirpe0xb9q8qiLsFr0=vr0=vr0dc8meaabaqaciaacaGaaeqabaqabeGadaaakeaadaWcaaqaaiabdcfaqnaaBaaaleaacqaIWaamaeqaaOGaeiikaGIaem4zaCMaeiiFaWhcciGae8hWdaNaeiykaKcabaGaemiuaa1aaSbaaSqaaiab=f8aYbqabaGccqGGOaakcqWGNbWzcqGG8baFcqWFapaCcqGGPaqkaaGaeyypa0ZaaSaaaeaacqWGXbqCdaqhaaWcbaGaemyyae2aaSbaaWqaaiabigdaXaqabaWccqWGJbWydaWgaaadbaGaeGymaedabeaaaSqaaiabcIcaOiabicdaWiabcMcaPaaakiabgwSixlabdghaXnaaDaaaleaacqWGHbqydaWgaaadbaGaeGOmaidabeaaliabdogaJnaaBaaameaacqaIYaGmaeqaaaWcbaGaeiikaGIaeGimaaJaeiykaKcaaOGaeyyXICTaeiOla4IaeiOla4IaeiOla4IaeyyXICTaemyCae3aa0baaSqaaiabdggaHnaaBaaameaacqWGUbGBaeqaaSGaem4yam2aaSbaaWqaaiabd6gaUbqabaaaleaacqGGOaakcqaIWaamcqGGPaqkaaaakeaacqWGXbqCdaqhaaWcbaGaemyyae2aaSbaaWqaaiabigdaXaqabaWccqWGJbWydaWgaaadbaGaeGymaedabeaaaSqaaiabcIcaOiab=f8aYjabcMcaPaaakiabgwSixlabdghaXnaaDaaaleaacqWGHbqydaWgaaadbaGaeGOmaidabeaaliabdogaJnaaBaaameaacqaIYaGmaeqaaaWcbaGaeiikaGIae8xWdiNaeiykaKcaaOGaeyyXICTaeiOla4IaeiOla4IaeiOla4IaeyyXICTaemyCae3aa0baaSqaaiabdggaHnaaBaaameaacqWGUbGBaeqaaSGaem4yam2aaSbaaWqaaiabd6gaUbqabaaaleaacqGGOaakcqWFbpGCcqGGPaqkaaaaaaaa@8E1C@

in the following way as a *scoring scheme*. The codon usage of *g *originating from G
 MathType@MTEF@5@5@+=feaafiart1ev1aaatCvAUfKttLearuWrP9MDH5MBPbIqV92AaeXatLxBI9gBamrtHrhAL1wy0L2yHvtyaeHbnfgDOvwBHrxAJfwnaebbnrfifHhDYfgasaacH8akY=wiFfYdH8Gipec8Eeeu0xXdbba9frFj0=OqFfea0dXdd9vqai=hGuQ8kuc9pgc9s8qqaq=dirpe0xb9q8qiLsFr0=vr0=vr0dc8meaabaqaciaacaGaaeqabaWaaeGaeaaakeaaimaacqWFge=raaa@382D@_0 _resembles more the prevalences of G
 MathType@MTEF@5@5@+=feaafiart1ev1aaatCvAUfKttLearuWrP9MDH5MBPbIqV92AaeXatLxBI9gBamrtHrhAL1wy0L2yHvtyaeHbnfgDOvwBHrxAJfwnaebbnrfifHhDYfgasaacH8akY=wiFfYdH8Gipec8Eeeu0xXdbba9frFj0=OqFfea0dXdd9vqai=hGuQ8kuc9pgc9s8qqaq=dirpe0xb9q8qiLsFr0=vr0=vr0dc8meaabaqaciaacaGaaeqabaWaaeGaeaaakeaaimaacqWFge=raaa@382D@_*ρ *_if

τρ,α>P0(g|π)Pρ(g|π).     (2)
 MathType@MTEF@5@5@+=feaafiart1ev1aaatCvAUfKttLearuWrP9MDH5MBPbIqV92AaeXatLxBI9gBaebbnrfifHhDYfgasaacH8akY=wiFfYdH8Gipec8Eeeu0xXdbba9frFj0=OqFfea0dXdd9vqai=hGuQ8kuc9pgc9s8qqaq=dirpe0xb9q8qiLsFr0=vr0=vr0dc8meaabaqaciaacaGaaeqabaqabeGadaaakeaaiiGacqWFepaDdaWgaaWcbaGae8xWdiNaeiilaWIae8xSdegabeaakiabg6da+maalaaabaGaemiuaa1aaSbaaSqaaiabicdaWaqabaGccqGGOaakcqWGNbWzcqGG8baFcqWFapaCcqGGPaqkaeaacqWGqbaudaWgaaWcbaGae8xWdihabeaakiabcIcaOiabdEgaNjabcYha8jab=b8aWjabcMcaPaaacqGGUaGlcaWLjaGaaCzcamaabmaabaGaeGOmaidacaGLOaGaayzkaaaaaa@4A87@

If this is the case for some *ρ *and if

ρ*=arg⁡min⁡ρ∈{1,2,...,r}P0(g|π)Pρ(g|π),
 MathType@MTEF@5@5@+=feaafiart1ev1aaatCvAUfKttLearuWrP9MDH5MBPbIqV92AaeXatLxBI9gBaebbnrfifHhDYfgasaacH8akY=wiFfYdH8Gipec8Eeeu0xXdbba9frFj0=OqFfea0dXdd9vqai=hGuQ8kuc9pgc9s8qqaq=dirpe0xb9q8qiLsFr0=vr0=vr0dc8meaabaqaciaacaGaaeqabaqabeGadaaakeaaiiGacqWFbpGCcqGGQaGkcqGH9aqpdaWfqaqaaiGbcggaHjabckhaYjabcEgaNjGbc2gaTjabcMgaPjabc6gaUbWcbaGae8xWdiNaeyicI4Saei4EaSNaeGymaeJaeiilaWIaeGOmaiJaeiilaWIaeiOla4IaeiOla4IaeiOla4IaeiilaWIaemOCaiNaeiyFa0habeaakmaalaaabaGaemiuaa1aaSbaaSqaaiabicdaWaqabaGccqGGOaakcqWGNbWzcqGG8baFcqWFapaCcqGGPaqkaeaacqWGqbaudaWgaaWcbaGae8xWdihabeaakiabcIcaOiabdEgaNjabcYha8jab=b8aWjabcMcaPaaacqGGSaalaaa@5A7B@

then gene *g *is considered to be pA originating from taxon Tρ*
 MathType@MTEF@5@5@+=feaafiart1ev1aaatCvAUfKttLearuWrP9MDH5MBPbIqV92AaeXatLxBI9gBamrtHrhAL1wy0L2yHvtyaeHbnfgDOvwBHrxAJfwnaebbnrfifHhDYfgasaacH8akY=wiFfYdH8Gipec8Eeeu0xXdbba9frFj0=OqFfea0dXdd9vqai=hGuQ8kuc9pgc9s8qqaq=dirpe0xb9q8qiLsFr0=vr0=vr0dc8meaabaqaciaacaGaaeqabaWaaeGaeaaakeaaimaacqWFtepvdaWgaaWcbaacciGae4xWdiNaeiOkaOcabeaaaaa@3B15@ represented by genome Gρ*
 MathType@MTEF@5@5@+=feaafiart1ev1aaatCvAUfKttLearuWrP9MDH5MBPbIqV92AaeXatLxBI9gBamrtHrhAL1wy0L2yHvtyaeHbnfgDOvwBHrxAJfwnaebbnrfifHhDYfgasaacH8akY=wiFfYdH8Gipec8Eeeu0xXdbba9frFj0=OqFfea0dXdd9vqai=hGuQ8kuc9pgc9s8qqaq=dirpe0xb9q8qiLsFr0=vr0=vr0dc8meaabaqaciaacaGaaeqabaWaaeGaeaaakeaaimaacqWFge=rdaWgaaWcbaacciGae4xWdiNaeiOkaOcabeaaaaa@3AFB@. This principle of deducing the putative donor has previously been introduced and validated [23].

How to choose the thresholds *τ*_*ρ*,*α *_needed in Equation 2? Let ℱπ
 MathType@MTEF@5@5@+=feaafiart1ev1aaatCvAUfKttLearuWrP9MDH5MBPbIqV92AaeXatLxBI9gBamrtHrhAL1wy0L2yHvtyaeHbnfgDOvwBHrxAJfwnaebbnrfifHhDYfgasaacH8akY=wiFfYdH8Gipec8Eeeu0xXdbba9frFj0=OqFfea0dXdd9vqai=hGuQ8kuc9pgc9s8qqaq=dirpe0xb9q8qiLsFr0=vr0=vr0dc8meaabaqaciaacaGaaeqabaWaaeGaeaaakeaaimaacqWFXeIrdaWgaaWcbaacciGae4hWdahabeaaaaa@3976@ be the set of all theoretically possible genes coding for protein *π*. For each *ρ *∈ {1, 2,..., *r*}, we consider the statistic

tρ(G):=ln⁡P0(G|π)Pρ(G|π),
 MathType@MTEF@5@5@+=feaafiart1ev1aaatCvAUfKttLearuWrP9MDH5MBPbIqV92AaeXatLxBI9gBaebbnrfifHhDYfgasaacH8akY=wiFfYdH8Gipec8Eeeu0xXdbba9frFj0=OqFfea0dXdd9vqai=hGuQ8kuc9pgc9s8qqaq=dirpe0xb9q8qiLsFr0=vr0=vr0dc8meaabaqaciaacaGaaeqabaqabeGadaaakeaacqWG0baDdaWgaaWcbaacciGae8xWdihabeaakiabcIcaOiabdEeahjabcMcaPiabcQda6iabg2da9iGbcYgaSjabc6gaUnaalaaabaGaemiuaa1aaSbaaSqaaiabicdaWaqabaGccqGGOaakcqWGhbWrcqGG8baFcqWFapaCcqGGPaqkaeaacqWGqbaudaWgaaWcbaGae8xWdihabeaakiabcIcaOiabdEeahjabcYha8jab=b8aWjabcMcaPaaacqGGSaalaaa@4A04@

where *G *∈ ℱπ
 MathType@MTEF@5@5@+=feaafiart1ev1aaatCvAUfKttLearuWrP9MDH5MBPbIqV92AaeXatLxBI9gBamrtHrhAL1wy0L2yHvtyaeHbnfgDOvwBHrxAJfwnaebbnrfifHhDYfgasaacH8akY=wiFfYdH8Gipec8Eeeu0xXdbba9frFj0=OqFfea0dXdd9vqai=hGuQ8kuc9pgc9s8qqaq=dirpe0xb9q8qiLsFr0=vr0=vr0dc8meaabaqaciaacaGaaeqabaWaaeGaeaaakeaaimaacqWFXeIrdaWgaaWcbaacciGae4hWdahabeaaaaa@3976@ is a random element distributed according to *P*_*ρ *_(· | *π*). Having computed the mean *μ*_*ρ *_and the standard deviation *σ*_*ρ *_of *t*_*ρ*_(*G*), we apply the central limit theorem: The random variable 1/*σ*_*ρ*_(*t*_*ρ*_(*G*) - *μ*_*ρ*_) is approximately distributed according to the standard normal distribution with the cumulative distribution function Φ. We determine the value *τ*_*ρ*, *α *_such that

α=1−Φ(ln⁡τρ,α−μρσρ).
 MathType@MTEF@5@5@+=feaafiart1ev1aaatCvAUfKttLearuWrP9MDH5MBPbIqV92AaeXatLxBI9gBaebbnrfifHhDYfgasaacH8akY=wiFfYdH8Gipec8Eeeu0xXdbba9frFj0=OqFfea0dXdd9vqai=hGuQ8kuc9pgc9s8qqaq=dirpe0xb9q8qiLsFr0=vr0=vr0dc8meaabaqaciaacaGaaeqabaqabeGadaaakeaaiiGacqWFXoqycqGH9aqpcqaIXaqmcqGHsislcqqHMoGrdaqadaqaamaalaaabaGagiiBaWMaeiOBa4Mae8hXdq3aaSbaaSqaaiab=f8aYjabcYcaSiab=f7aHbqabaGccqGHsislcqWF8oqBdaWgaaWcbaGae8xWdihabeaaaOqaaiab=n8aZnaaBaaaleaacqWFbpGCaeqaaaaaaOGaayjkaiaawMcaaiabc6caUaaa@465B@

The parameter *α *serves as SIGI-HMM's sensitivity controller. It can be adjusted by the user. Please note that the impact of parameter *α *onto the decision is independent of G
 MathType@MTEF@5@5@+=feaafiart1ev1aaatCvAUfKttLearuWrP9MDH5MBPbIqV92AaeXatLxBI9gBamrtHrhAL1wy0L2yHvtyaeHbnfgDOvwBHrxAJfwnaebbnrfifHhDYfgasaacH8akY=wiFfYdH8Gipec8Eeeu0xXdbba9frFj0=OqFfea0dXdd9vqai=hGuQ8kuc9pgc9s8qqaq=dirpe0xb9q8qiLsFr0=vr0=vr0dc8meaabaqaciaacaGaaeqabaWaaeGaeaaakeaaimaacqWFge=raaa@382D@_0 _and G
 MathType@MTEF@5@5@+=feaafiart1ev1aaatCvAUfKttLearuWrP9MDH5MBPbIqV92AaeXatLxBI9gBamrtHrhAL1wy0L2yHvtyaeHbnfgDOvwBHrxAJfwnaebbnrfifHhDYfgasaacH8akY=wiFfYdH8Gipec8Eeeu0xXdbba9frFj0=OqFfea0dXdd9vqai=hGuQ8kuc9pgc9s8qqaq=dirpe0xb9q8qiLsFr0=vr0=vr0dc8meaabaqaciaacaGaaeqabaWaaeGaeaaakeaaimaacqWFge=raaa@382D@_*ρ*_.

### Eliminating putatively highly expressed genes

In several genomes, highly expressed genes show a specific codon usage which deviates from the average one and resembles codon prevalences observed in genes coding for ribosomal proteins; see e.g. [8]. We name these genes *putatively highly expressed *(PHX). On the one hand, it is unlikely that these genes were acquired *via *HGT. On the other hand, methods based on codon usage tend to classify them as pA. This needs to be prevented explicitly. We use an approach similar to the GCB score introduced in [30]. It was shown that this methods is one of the best to predict gene expressivity [31]. Let qac(0,rib)
 MathType@MTEF@5@5@+=feaafiart1ev1aaatCvAUfKttLearuWrP9MDH5MBPbIqV92AaeXatLxBI9gBaebbnrfifHhDYfgasaacH8akY=wiFfYdH8Gipec8Eeeu0xXdbba9frFj0=OqFfea0dXdd9vqai=hGuQ8kuc9pgc9s8qqaq=dirpe0xb9q8qiLsFr0=vr0=vr0dc8meaabaqaciaacaGaaeqabaqabeGadaaakeaacqWGXbqCdaqhaaWcbaGaemyyaeMaem4yamgabaGaeiikaGIaeGimaaJaeiilaWIaeeOCaiNaeeyAaKMaeeOyaiMaeiykaKcaaaaa@386D@ be the synonymous codon frequencies for the ribosomal genes of genome G
 MathType@MTEF@5@5@+=feaafiart1ev1aaatCvAUfKttLearuWrP9MDH5MBPbIqV92AaeXatLxBI9gBamrtHrhAL1wy0L2yHvtyaeHbnfgDOvwBHrxAJfwnaebbnrfifHhDYfgasaacH8akY=wiFfYdH8Gipec8Eeeu0xXdbba9frFj0=OqFfea0dXdd9vqai=hGuQ8kuc9pgc9s8qqaq=dirpe0xb9q8qiLsFr0=vr0=vr0dc8meaabaqaciaacaGaaeqabaWaaeGaeaaakeaaimaacqWFge=raaa@382D@_0 _and let

P0,rib(g|π):=qa1c1(0,rib)⋅qa2c2(0,rib)⋅...⋅qa3c3(0,rib).     (3)
 MathType@MTEF@5@5@+=feaafiart1ev1aaatCvAUfKttLearuWrP9MDH5MBPbIqV92AaeXatLxBI9gBaebbnrfifHhDYfgasaacH8akY=wiFfYdH8Gipec8Eeeu0xXdbba9frFj0=OqFfea0dXdd9vqai=hGuQ8kuc9pgc9s8qqaq=dirpe0xb9q8qiLsFr0=vr0=vr0dc8meaabaqaciaacaGaaeqabaqabeGadaaakeaacqWGqbaudaWgaaWcbaGaeGimaaJaeiilaWIaeeOCaiNaeeyAaKMaeeOyaigabeaakiabcIcaOiabdEgaNjabcYha8HGaciab=b8aWjabcMcaPiabcQda6iabg2da9iabdghaXnaaDaaaleaacqWGHbqydaWgaaadbaGaeGymaedabeaaliabdogaJnaaBaaameaacqaIXaqmaeqaaaWcbaGaeiikaGIaeGimaaJaeiilaWIaeeOCaiNaeeyAaKMaeeOyaiMaeiykaKcaaOGaeyyXICTaemyCae3aa0baaSqaaiabdggaHnaaBaaameaacqaIYaGmaeqaaSGaem4yam2aaSbaaWqaaiabikdaYaqabaaaleaacqGGOaakcqaIWaamcqGGSaalcqqGYbGCcqqGPbqAcqqGIbGycqGGPaqkaaGccqGHflY1cqGGUaGlcqGGUaGlcqGGUaGlcqGHflY1cqWGXbqCdaqhaaWcbaGaemyyae2aaSbaaWqaaiabiodaZaqabaWccqWGJbWydaWgaaadbaGaeG4mamdabeaaaSqaaiabcIcaOiabicdaWiabcYcaSiabbkhaYjabbMgaPjabbkgaIjabcMcaPaaakiabc6caUiaaxMaacaWLjaWaaeWaaeaacqaIZaWmaiaawIcacaGLPaaaaaa@74C3@

If

trib(g):=ln⁡P0,rib(g|π)Pρ(g|π)>θ,
 MathType@MTEF@5@5@+=feaafiart1ev1aaatCvAUfKttLearuWrP9MDH5MBPbIqV92AaeXatLxBI9gBaebbnrfifHhDYfgasaacH8akY=wiFfYdH8Gipec8Eeeu0xXdbba9frFj0=OqFfea0dXdd9vqai=hGuQ8kuc9pgc9s8qqaq=dirpe0xb9q8qiLsFr0=vr0=vr0dc8meaabaqaciaacaGaaeqabaqabeGadaaakeaacqWG0baDdaWgaaWcbaGaeeOCaiNaeeyAaKMaeeOyaigabeaakiabcIcaOiabdEgaNjabcMcaPiabcQda6iabg2da9iGbcYgaSjabc6gaUnaalaaabaGaemiuaa1aaSbaaSqaaiabicdaWiabcYcaSiabbkhaYjabbMgaPjabbkgaIbqabaGccqGGOaakcqWGNbWzcqGG8baFiiGacqWFapaCcqGGPaqkaeaacqWGqbaudaWgaaWcbaGae8xWdihabeaakiabcIcaOiabdEgaNjabcYha8jab=b8aWjabcMcaPaaacqGH+aGpcqWF4oqCcqGGSaalaaa@54C0@

we consider the gene *g *as not alien (see [2,8]).

The threshold *θ *is determined as follows: Let *μ*_0 _and *μ*_0,rib _be the mean values and *σ*_0 _and *σ*_0,rib _be the standard deviations of the test statistic *t*_rib_(*G*), where *G *is distributed according to *P*_0_(· | *π*) and *P*_0,rib_(· | *π*), respectively. The distribution functions of 1/*σ*_0_(*t*_rib_(*G*) - *μ*_0_) and 1/*σ*_0,rib_(*t*_rib_(*G*) - *μ*_0,rib_) are approximately standard normal. We choose *θ *in such a way that

1−Φ(θ−μ0σ0)=Φ(θ−μ0,ribσ0,rib).     (4)
 MathType@MTEF@5@5@+=feaafiart1ev1aaatCvAUfKttLearuWrP9MDH5MBPbIqV92AaeXatLxBI9gBaebbnrfifHhDYfgasaacH8akY=wiFfYdH8Gipec8Eeeu0xXdbba9frFj0=OqFfea0dXdd9vqai=hGuQ8kuc9pgc9s8qqaq=dirpe0xb9q8qiLsFr0=vr0=vr0dc8meaabaqaciaacaGaaeqabaqabeGadaaakeaacqaIXaqmcqGHsislcqqHMoGrdaqadaqaamaalaaabaacciGae8hUdeNaeyOeI0Iae8hVd02aaSbaaSqaaiabicdaWaqabaaakeaacqWFdpWCdaWgaaWcbaGaeGimaadabeaaaaaakiaawIcacaGLPaaacqGH9aqpcqqHMoGrdaqadaqaamaalaaabaGae8hUdeNaeyOeI0Iae8hVd02aaSbaaSqaaiabicdaWiabcYcaSiabbkhaYjabbMgaPjabbkgaIbqabaaakeaacqWFdpWCdaWgaaWcbaGaeGimaaJaeiilaWIaeeOCaiNaeeyAaKMaeeOyaigabeaaaaaakiaawIcacaGLPaaacqGGUaGlcaWLjaGaaCzcamaabmaabaGaeGinaqdacaGLOaGaayzkaaaaaa@54F0@

Thus, the error of the first and second kind are of equal size.

### Architecture of the HMM

**Figure 1:**
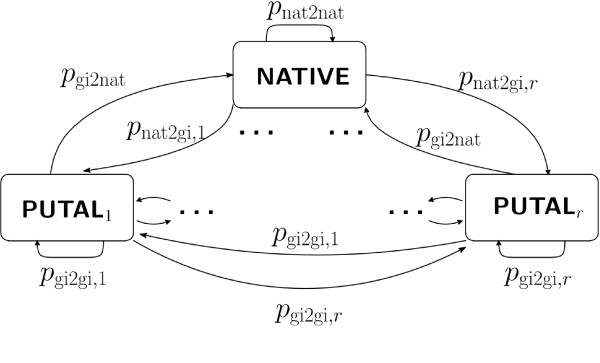
States and transition probabilities of SIGI-HMM's Markov chain. The state NATIVE represents genes which are unsuspicious with respect to synonymous codon frequencies. For *ρ *= 1, 2,..., *r*, the state PUTAL_ρ _models genes, whose codon usage resembles more the prevalences of genomes G
 MathType@MTEF@5@5@+=feaafiart1ev1aaatCvAUfKttLearuWrP9MDH5MBPbIqV92AaeXatLxBI9gBamrtHrhAL1wy0L2yHvtyaeHbnfgDOvwBHrxAJfwnaebbnrfifHhDYfgasaacH8akY=wiFfYdH8Gipec8Eeeu0xXdbba9frFj0=OqFfea0dXdd9vqai=hGuQ8kuc9pgc9s8qqaq=dirpe0xb9q8qiLsFr0=vr0=vr0dc8meaabaqaciaacaGaaeqabaWaaeGaeaaakeaaimaacqWFge=raaa@382D@_*ρ *_which represents taxon T
 MathType@MTEF@5@5@+=feaafiart1ev1aaatCvAUfKttLearuWrP9MDH5MBPbIqV92AaeXatLxBI9gBamrtHrhAL1wy0L2yHvtyaeHbnfgDOvwBHrxAJfwnaebbnrfifHhDYfgasaacH8akY=wiFfYdH8Gipec8Eeeu0xXdbba9frFj0=OqFfea0dXdd9vqai=hGuQ8kuc9pgc9s8qqaq=dirpe0xb9q8qiLsFr0=vr0=vr0dc8meaabaqaciaacaGaaeqabaWaaeGaeaaakeaaimaacqWFtepvaaa@3847@_*ρ*_. Each transition from state *x *to state *y *is characterized by its transition probability *p*_*x*2*y*_. In order to model the mosaic structure of GI composition, transitions from any state PUTAL_*ρ *_to any other one PUTAL_*σ *_are allowed.

Figure 1 depicts the architecture of the implemented HMM. The state NATIVE corresponds to genes having an unsuspicious codon usage. The states PUTAL_1_, PUTAL_2_,..., PUTAL_*r *_represent putatively alien genes originating from taxa T
 MathType@MTEF@5@5@+=feaafiart1ev1aaatCvAUfKttLearuWrP9MDH5MBPbIqV92AaeXatLxBI9gBamrtHrhAL1wy0L2yHvtyaeHbnfgDOvwBHrxAJfwnaebbnrfifHhDYfgasaacH8akY=wiFfYdH8Gipec8Eeeu0xXdbba9frFj0=OqFfea0dXdd9vqai=hGuQ8kuc9pgc9s8qqaq=dirpe0xb9q8qiLsFr0=vr0=vr0dc8meaabaqaciaacaGaaeqabaWaaeGaeaaakeaaimaacqWFtepvaaa@3847@_1 _to T
 MathType@MTEF@5@5@+=feaafiart1ev1aaatCvAUfKttLearuWrP9MDH5MBPbIqV92AaeXatLxBI9gBamrtHrhAL1wy0L2yHvtyaeHbnfgDOvwBHrxAJfwnaebbnrfifHhDYfgasaacH8akY=wiFfYdH8Gipec8Eeeu0xXdbba9frFj0=OqFfea0dXdd9vqai=hGuQ8kuc9pgc9s8qqaq=dirpe0xb9q8qiLsFr0=vr0=vr0dc8meaabaqaciaacaGaaeqabaWaaeGaeaaakeaaimaacqWFtepvaaa@3847@_*r*_. GIs frequently have a mosaic structure which is due to their generation in a multistep process (see [2]). Therefore, we allow transitions from any PUTAL (i.e. donor) state to any other one.

In order to implement our sensitivity controller, we let the transition probabilities depend on the protein under consideration. Thus, the Markov chain presented in Figure 1 is in fact an inhomogeneous one driven by the series  _0 _of proteins encoded by G
 MathType@MTEF@5@5@+=feaafiart1ev1aaatCvAUfKttLearuWrP9MDH5MBPbIqV92AaeXatLxBI9gBamrtHrhAL1wy0L2yHvtyaeHbnfgDOvwBHrxAJfwnaebbnrfifHhDYfgasaacH8akY=wiFfYdH8Gipec8Eeeu0xXdbba9frFj0=OqFfea0dXdd9vqai=hGuQ8kuc9pgc9s8qqaq=dirpe0xb9q8qiLsFr0=vr0=vr0dc8meaabaqaciaacaGaaeqabaWaaeGaeaaakeaaimaacqWFge=raaa@382D@_0_. To simplify notation, we have omitted the index *π*, which refers to the protein. Instead, we identify a protein by its index originating from  _0_.

Solving some linear equations, the transition probabilities given in Figure 1 can be determined in such a way that

a⋅τρ,α=pgi2gi,ρpgi2naandb⋅τρ,α=pna2gi,ρpna2na.
 MathType@MTEF@5@5@+=feaafiart1ev1aaatCvAUfKttLearuWrP9MDH5MBPbIqV92AaeXatLxBI9gBaebbnrfifHhDYfgasaacH8akY=wiFfYdH8Gipec8Eeeu0xXdbba9frFj0=OqFfea0dXdd9vqai=hGuQ8kuc9pgc9s8qqaq=dirpe0xb9q8qiLsFr0=vr0=vr0dc8meaabaqaciaacaGaaeqabaqabeGadaaakeaafaqabeqadaaabaGaemyyaeMaeyyXICncciGae8hXdq3aaSbaaSqaaiab=f8aYjabcYcaSiab=f7aHbqabaGccqGH9aqpdaWcaaqaaiabdchaWnaaBaaaleaacqqGNbWzcqqGPbqAcqqGYaGmcqqGNbWzcqqGPbqAcqGGSaalcqWFbpGCaeqaaaGcbaGaemiCaa3aaSbaaSqaaiabbEgaNjabbMgaPjabbkdaYiabb6gaUjabbggaHbqabaaaaaGcbaGaeeyyaeMaeeOBa4MaeeizaqgabaGaemOyaiMaeyyXICTae8hXdq3aaSbaaSqaaiab=f8aYjabcYcaSiab=f7aHbqabaGccqGH9aqpdaWcaaqaaiabdchaWnaaBaaaleaacqqGUbGBcqqGHbqycqqGYaGmcqqGNbWzcqqGPbqAcqGGSaalcqWFbpGCaeqaaaGcbaGaemiCaa3aaSbaaSqaaiabb6gaUjabbggaHjabbkdaYiabb6gaUjabbggaHbqabaaaaaaakiabc6caUaaa@6C1A@

*a *and *b *are positive constants which were chosen appropriately to generate GIs which are at mean shorter than the surrounding regions of native genes. The probabilities *p*_*x*2*y *_correspond to transitions from state *x *to *y *(see Figure 1).

We extend the Markov chain *X*_1_, *X*_2_, ..., *X*_ℓ _driven by the state diagram given in Figure 1 to a HMM *X*_1_, *Y*_1_, *X*_2_, *Y*_2 _..., *X*_ℓ_, *Y*_ℓ _in the following way: For *π *= 1, 2..., ℓ, the random *emission Y*_*π *_takes values in the sample space ℱπ
 MathType@MTEF@5@5@+=feaafiart1ev1aaatCvAUfKttLearuWrP9MDH5MBPbIqV92AaeXatLxBI9gBamrtHrhAL1wy0L2yHvtyaeHbnfgDOvwBHrxAJfwnaebbnrfifHhDYfgasaacH8akY=wiFfYdH8Gipec8Eeeu0xXdbba9frFj0=OqFfea0dXdd9vqai=hGuQ8kuc9pgc9s8qqaq=dirpe0xb9q8qiLsFr0=vr0=vr0dc8meaabaqaciaacaGaaeqabaWaaeGaeaaakeaaimaacqWFXeIrdaWgaaWcbaacciGae4hWdahabeaaaaa@3976@ defined above. For *ρ *= 1, 2,..., *r*, the *emission probabilities *are defined by means of Equation 1 as follows:

*P*(*Y*_*π *_= *g *| *X*_*π *_= NATIVE) = *P*_0_(*g *| *π*)     and     *P*(*Y*_*π *_= *g *| *X*_*π *_= PUTAL_*ρ*_) = *P*_*ρ*_(*g *| *π*).     (5)

As already explained, PHX genes have to be eliminated. Our test for putatively highly expressed genes classifies genes as phx or ¬phx. In order to integrate these predictions into the HMM, we interpret the outputs as a random sequence *H*_1_, *H*_2_,..., *H*_ℓ _of *hints*. Please note that an emission is now a combination of a gene and a hint. Hints are interpreted the following way: For the native state we define

P(Hπ=phx|Xπ=NATIVE,Yπ=gπ)={1if trib(gπ)>θ;0otherwise.
 MathType@MTEF@5@5@+=feaafiart1ev1aaatCvAUfKttLearuWrP9MDH5MBPbIqV92AaeXatLxBI9gBaebbnrfifHhDYfgasaacH8akY=wiFfYdH8Gipec8Eeeu0xXdbba9frFj0=OqFfea0dXdd9vqai=hGuQ8kuc9pgc9s8qqaq=dirpe0xb9q8qiLsFr0=vr0=vr0dc8meaabaqaciaacaGaaeqabaqabeGadaaakeaacqWGqbaucqGGOaakcqWGibasdaWgaaWcbaacciGae8hWdahabeaakiabg2da9iabbchaWjabbIgaOjabbIha4jabcYha8jabdIfaynaaBaaaleaacqWFapaCaeqaaOGaeyypa0JaeeOta4KaeeyqaeKaeeivaqLaeeysaKKaeeOvayLaeeyrauKaeiilaWIaemywaK1aaSbaaSqaaiab=b8aWbqabaGccqGH9aqpcqWGNbWzdaWgaaWcbaGae8hWdahabeaakiabcMcaPiabg2da9maaceaabaqbaeaabiGaaaqaaiabigdaXaqaaiabbMgaPjabbAgaMjaaykW7cqWG0baDdaWgaaWcbaGaeeOCaiNaeeyAaKMaeeOyaigabeaakiabcIcaOiabdEgaNnaaBaaaleaacqWFapaCaeqaaOGaeiykaKIaeyOpa4Jae8hUdeNaei4oaSdabaGaeGimaadabaGaee4Ba8MaeeiDaqNaeeiAaGMaeeyzauMaeeOCaiNaee4DaCNaeeyAaKMaee4CamNaeeyzauMaeiOla4caaaGaay5Eaaaaaa@708D@

For *ρ *= 1, 2,..., *r*, the *emission probability *given a pA state is defined by

*P*(*H*_*π *_= ¬phx | *X*_*π *_∈ {PUTAL_1_, PUTAL_2_, ..., PUTAL_*r*_}) = 1.

It is biological evidence, which led to the above definitions. The products of highly expressed genes are involved in complex interactions. Therefore, it is highly unlikely that these genes can be replaced by HGT. Please note that the algorithm has – due to our design – to consider each hint.

### Determination of the codon-specific core and atypical genes

It might be that some pA genes originate from sources not characterized by our set of putative donors (see below). In order to identify these atypical genes, we determine the *codon-specific core *(*CSC*) of a genome, which consists of those genes having an unsuspicious codon usage. Having chosen a protein *π *∈ _0 _and the related gene *g *∈ G
 MathType@MTEF@5@5@+=feaafiart1ev1aaatCvAUfKttLearuWrP9MDH5MBPbIqV92AaeXatLxBI9gBamrtHrhAL1wy0L2yHvtyaeHbnfgDOvwBHrxAJfwnaebbnrfifHhDYfgasaacH8akY=wiFfYdH8Gipec8Eeeu0xXdbba9frFj0=OqFfea0dXdd9vqai=hGuQ8kuc9pgc9s8qqaq=dirpe0xb9q8qiLsFr0=vr0=vr0dc8meaabaqaciaacaGaaeqabaWaaeGaeaaakeaaimaacqWFge=raaa@382D@_0_, we consider a random element *G *of the set ℱπ
 MathType@MTEF@5@5@+=feaafiart1ev1aaatCvAUfKttLearuWrP9MDH5MBPbIqV92AaeXatLxBI9gBamrtHrhAL1wy0L2yHvtyaeHbnfgDOvwBHrxAJfwnaebbnrfifHhDYfgasaacH8akY=wiFfYdH8Gipec8Eeeu0xXdbba9frFj0=OqFfea0dXdd9vqai=hGuQ8kuc9pgc9s8qqaq=dirpe0xb9q8qiLsFr0=vr0=vr0dc8meaabaqaciaacaGaaeqabaWaaeGaeaaakeaaimaacqWFXeIrdaWgaaWcbaacciGae4hWdahabeaaaaa@3976@ distributed according to *P*_0_(· | *π*) (see Equation 1). For the following test, we identifed those amino acids *a *encoded by more than one codon and occurring at least 5 times (*n*_*a *_≥ 5) in the protein. For each codon *c *which encodes amino acid *a *we introduce a random variable count_*c*_(*G*) = #*c*, which follows a binomial distribution characterized by the expected value naqac(0)
 MathType@MTEF@5@5@+=feaafiart1ev1aaatCvAUfKttLearuWrP9MDH5MBPbIqV92AaeXatLxBI9gBaebbnrfifHhDYfgasaacH8akY=wiFfYdH8Gipec8Eeeu0xXdbba9frFj0=OqFfea0dXdd9vqai=hGuQ8kuc9pgc9s8qqaq=dirpe0xb9q8qiLsFr0=vr0=vr0dc8meaabaqaciaacaGaaeqabaqabeGadaaakeaacqWGUbGBdaWgaaWcbaGaemyyaegabeaakiabdghaXnaaDaaaleaacqWGHbqycqWGJbWyaeaacqGGOaakcqaIWaamcqGGPaqkaaaaaa@3664@ and variance naqac(0)
 MathType@MTEF@5@5@+=feaafiart1ev1aaatCvAUfKttLearuWrP9MDH5MBPbIqV92AaeXatLxBI9gBaebbnrfifHhDYfgasaacH8akY=wiFfYdH8Gipec8Eeeu0xXdbba9frFj0=OqFfea0dXdd9vqai=hGuQ8kuc9pgc9s8qqaq=dirpe0xb9q8qiLsFr0=vr0=vr0dc8meaabaqaciaacaGaaeqabaqabeGadaaakeaacqWGUbGBdaWgaaWcbaGaemyyaegabeaakiabdghaXnaaDaaaleaacqWGHbqycqWGJbWyaeaacqGGOaakcqaIWaamcqGGPaqkaaaaaa@3664@(1 - qac(0)
 MathType@MTEF@5@5@+=feaafiart1ev1aaatCvAUfKttLearuWrP9MDH5MBPbIqV92AaeXatLxBI9gBaebbnrfifHhDYfgasaacH8akY=wiFfYdH8Gipec8Eeeu0xXdbba9frFj0=OqFfea0dXdd9vqai=hGuQ8kuc9pgc9s8qqaq=dirpe0xb9q8qiLsFr0=vr0=vr0dc8meaabaqaciaacaGaaeqabaqabeGadaaakeaacqWGUbGBdaWgaaWcbaGaemyyaegabeaakiabdghaXnaaDaaaleaacqWGHbqycqWGJbWyaeaacqGGOaakcqaIWaamcqGGPaqkaaaaaa@3664@). The statistic

φ c(G):=countc(G)−naqac(0)naqac(0)(1−qac(0))
 MathType@MTEF@5@5@+=feaafiart1ev1aaatCvAUfKttLearuWrP9MDH5MBPbIqV92AaeXatLxBI9gBaebbnrfifHhDYfgasaacH8akY=wiFfYdH8Gipec8Eeeu0xXdbba9frFj0=OqFfea0dXdd9vqai=hGuQ8kuc9pgc9s8qqaq=dirpe0xb9q8qiLsFr0=vr0=vr0dc8meaabaqaciaacaGaaeqabaqabeGadaaakeaaiiGacqWFgpGzdaWgaaWcbaGaaGPaVlabdogaJbqabaGccqGGOaakcqWGhbWrcqGGPaqkcqGG6aGocqGH9aqpdaWcaaqaaiabbogaJjabb+gaVjabbwha1jabb6gaUjabbsha0naaBaaaleaacqWGJbWyaeqaaOGaeiikaGIaem4raCKaeiykaKIaeyOeI0IaemOBa42aaSbaaSqaaiabdggaHbqabaGccqWGXbqCdaqhaaWcbaGaemyyaeMaem4yamgabaGaeiikaGIaeGimaaJaeiykaKcaaaGcbaWaaOaaaeaacqWGUbGBdaWgaaWcbaGaemyyaegabeaakiabdghaXnaaDaaaleaacqWGHbqycqWGJbWyaeaacqGGOaakcqaIWaamcqGGPaqkaaGccqGGOaakcqaIXaqmcqGHsislcqWGXbqCdaqhaaWcbaGaemyyaeMaem4yamgabaGaeiikaGIaeGimaaJaeiykaKcaaOGaeiykaKcaleqaaaaaaaa@6091@

is approximately distributed according to the standard normal distribution. For each *δ *∈ (0, 1) there is exactly one *θ*_*δ *_> 0 such that

P(|φ c(G)|  ≥θδ)=2Φ(−θδ)=δγ,
 MathType@MTEF@5@5@+=feaafiart1ev1aaatCvAUfKttLearuWrP9MDH5MBPbIqV92AaeXatLxBI9gBaebbnrfifHhDYfgasaacH8akY=wiFfYdH8Gipec8Eeeu0xXdbba9frFj0=OqFfea0dXdd9vqai=hGuQ8kuc9pgc9s8qqaq=dirpe0xb9q8qiLsFr0=vr0=vr0dc8meaabaqaciaacaGaaeqabaqabeGadaaakeaacqWGqbaucqGGOaakcqGG8baFiiGacqWFgpGzdaWgaaWcbaGaaGPaVlabdogaJbqabaGccqGGOaakcqWGhbWrcqGGPaqkcqGG8baFcaaMc8UaaGPaVlabgwMiZkab=H7aXnaaBaaaleaacqWF0oazaeqaaOGaeiykaKIaeyypa0JaeGOmaiJaeuOPdyKaeiikaGIaeyOeI0Iae8hUde3aaSbaaSqaaiab=r7aKbqabaGccqGGPaqkcqGH9aqpdaWcaaqaaiab=r7aKbqaaiab=n7aNbaacqGGSaalaaa@5153@

where *γ *is the occurrence of those amino acids considered in this section. In analogy to [32], we name the gene *g δ-typical *(*δ *∈ (0, 1)), if for all codons *c*

|*φ*_*c*_(*g*)| <*θ*_*δ*_.

This is why the probability of being not *δ*-typical is for a random gene G less than or equal to *δ*. Setting *δ *to 10/ℓ, where ℓ is the number of G
 MathType@MTEF@5@5@+=feaafiart1ev1aaatCvAUfKttLearuWrP9MDH5MBPbIqV92AaeXatLxBI9gBamrtHrhAL1wy0L2yHvtyaeHbnfgDOvwBHrxAJfwnaebbnrfifHhDYfgasaacH8akY=wiFfYdH8Gipec8Eeeu0xXdbba9frFj0=OqFfea0dXdd9vqai=hGuQ8kuc9pgc9s8qqaq=dirpe0xb9q8qiLsFr0=vr0=vr0dc8meaabaqaciaacaGaaeqabaWaaeGaeaaakeaaimaacqWFge=raaa@382D@_0_'s genes, turned out to be adequate. Only few genes (< 1%) were labelled as atypical (see Results). Therefore, the exact value of *δ *is uncritical. This observation confirms that our selection of codon usage tables covers the prevalences of putative donors to a great extent.

The algorithm for computing the CSC of genome G
 MathType@MTEF@5@5@+=feaafiart1ev1aaatCvAUfKttLearuWrP9MDH5MBPbIqV92AaeXatLxBI9gBamrtHrhAL1wy0L2yHvtyaeHbnfgDOvwBHrxAJfwnaebbnrfifHhDYfgasaacH8akY=wiFfYdH8Gipec8Eeeu0xXdbba9frFj0=OqFfea0dXdd9vqai=hGuQ8kuc9pgc9s8qqaq=dirpe0xb9q8qiLsFr0=vr0=vr0dc8meaabaqaciaacaGaaeqabaWaaeGaeaaakeaaimaacqWFge=raaa@382D@_0 _first removes all genes from G
 MathType@MTEF@5@5@+=feaafiart1ev1aaatCvAUfKttLearuWrP9MDH5MBPbIqV92AaeXatLxBI9gBamrtHrhAL1wy0L2yHvtyaeHbnfgDOvwBHrxAJfwnaebbnrfifHhDYfgasaacH8akY=wiFfYdH8Gipec8Eeeu0xXdbba9frFj0=OqFfea0dXdd9vqai=hGuQ8kuc9pgc9s8qqaq=dirpe0xb9q8qiLsFr0=vr0=vr0dc8meaabaqaciaacaGaaeqabaWaaeGaeaaakeaaimaacqWFge=raaa@382D@_0 _that are not *δ*-typical. Then the synonymous codon frequencies of the remaining genes G
 MathType@MTEF@5@5@+=feaafiart1ev1aaatCvAUfKttLearuWrP9MDH5MBPbIqV92AaeXatLxBI9gBamrtHrhAL1wy0L2yHvtyaeHbnfgDOvwBHrxAJfwnaebbnrfifHhDYfgasaacH8akY=wiFfYdH8Gipec8Eeeu0xXdbba9frFj0=OqFfea0dXdd9vqai=hGuQ8kuc9pgc9s8qqaq=dirpe0xb9q8qiLsFr0=vr0=vr0dc8meaabaqaciaacaGaaeqabaWaaeGaeaaakeaaimaacqWFge=raaa@382D@_typ _are recomputed and the genes not *δ*-typical with respect to the new frequencies are removed from G
 MathType@MTEF@5@5@+=feaafiart1ev1aaatCvAUfKttLearuWrP9MDH5MBPbIqV92AaeXatLxBI9gBamrtHrhAL1wy0L2yHvtyaeHbnfgDOvwBHrxAJfwnaebbnrfifHhDYfgasaacH8akY=wiFfYdH8Gipec8Eeeu0xXdbba9frFj0=OqFfea0dXdd9vqai=hGuQ8kuc9pgc9s8qqaq=dirpe0xb9q8qiLsFr0=vr0=vr0dc8meaabaqaciaacaGaaeqabaWaaeGaeaaakeaaimaacqWFge=raaa@382D@_*typ*_. This is done as long as there are such genes in G
 MathType@MTEF@5@5@+=feaafiart1ev1aaatCvAUfKttLearuWrP9MDH5MBPbIqV92AaeXatLxBI9gBamrtHrhAL1wy0L2yHvtyaeHbnfgDOvwBHrxAJfwnaebbnrfifHhDYfgasaacH8akY=wiFfYdH8Gipec8Eeeu0xXdbba9frFj0=OqFfea0dXdd9vqai=hGuQ8kuc9pgc9s8qqaq=dirpe0xb9q8qiLsFr0=vr0=vr0dc8meaabaqaciaacaGaaeqabaWaaeGaeaaakeaaimaacqWFge=raaa@382D@_*typ*_. Our experiments showed that this algorithm converged for all completely sequenced genomes to a CSC G
 MathType@MTEF@5@5@+=feaafiart1ev1aaatCvAUfKttLearuWrP9MDH5MBPbIqV92AaeXatLxBI9gBamrtHrhAL1wy0L2yHvtyaeHbnfgDOvwBHrxAJfwnaebbnrfifHhDYfgasaacH8akY=wiFfYdH8Gipec8Eeeu0xXdbba9frFj0=OqFfea0dXdd9vqai=hGuQ8kuc9pgc9s8qqaq=dirpe0xb9q8qiLsFr0=vr0=vr0dc8meaabaqaciaacaGaaeqabaWaaeGaeaaakeaaimaacqWFge=raaa@382D@_*typ *_containing at least 75% of all genes. The *atypical *genes are those not contained in the CSC G
 MathType@MTEF@5@5@+=feaafiart1ev1aaatCvAUfKttLearuWrP9MDH5MBPbIqV92AaeXatLxBI9gBamrtHrhAL1wy0L2yHvtyaeHbnfgDOvwBHrxAJfwnaebbnrfifHhDYfgasaacH8akY=wiFfYdH8Gipec8Eeeu0xXdbba9frFj0=OqFfea0dXdd9vqai=hGuQ8kuc9pgc9s8qqaq=dirpe0xb9q8qiLsFr0=vr0=vr0dc8meaabaqaciaacaGaaeqabaWaaeGaeaaakeaaimaacqWFge=raaa@382D@_*typ*_.

### Predicting genomic islands

Using the Viterbi algorithm (see e.g. [33,34]), SIGI-HMM computes at first the Viterbi path (i.e. the most probable sequence of states). All genes labeled as atypical and all genes assigned to one of the states PUTAL_*ρ *_(*ρ *= 1, 2, ..., *r*) are considered as belonging to GIs. Since it is reasonable to expect inside GIs genes with a codon usage similar to native ones, GIs separated by less than four native genes can optionally be merged. This merging distance can be set by the user.

### Selecting putative donors

For each genome G
 MathType@MTEF@5@5@+=feaafiart1ev1aaatCvAUfKttLearuWrP9MDH5MBPbIqV92AaeXatLxBI9gBamrtHrhAL1wy0L2yHvtyaeHbnfgDOvwBHrxAJfwnaebbnrfifHhDYfgasaacH8akY=wiFfYdH8Gipec8Eeeu0xXdbba9frFj0=OqFfea0dXdd9vqai=hGuQ8kuc9pgc9s8qqaq=dirpe0xb9q8qiLsFr0=vr0=vr0dc8meaabaqaciaacaGaaeqabaWaaeGaeaaakeaaimaacqWFge=raaa@382D@_0_, an individual set of putative donors G
 MathType@MTEF@5@5@+=feaafiart1ev1aaatCvAUfKttLearuWrP9MDH5MBPbIqV92AaeXatLxBI9gBamrtHrhAL1wy0L2yHvtyaeHbnfgDOvwBHrxAJfwnaebbnrfifHhDYfgasaacH8akY=wiFfYdH8Gipec8Eeeu0xXdbba9frFj0=OqFfea0dXdd9vqai=hGuQ8kuc9pgc9s8qqaq=dirpe0xb9q8qiLsFr0=vr0=vr0dc8meaabaqaciaacaGaaeqabaWaaeGaeaaakeaaimaacqWFge=raaa@382D@_1_, G
 MathType@MTEF@5@5@+=feaafiart1ev1aaatCvAUfKttLearuWrP9MDH5MBPbIqV92AaeXatLxBI9gBamrtHrhAL1wy0L2yHvtyaeHbnfgDOvwBHrxAJfwnaebbnrfifHhDYfgasaacH8akY=wiFfYdH8Gipec8Eeeu0xXdbba9frFj0=OqFfea0dXdd9vqai=hGuQ8kuc9pgc9s8qqaq=dirpe0xb9q8qiLsFr0=vr0=vr0dc8meaabaqaciaacaGaaeqabaWaaeGaeaaakeaaimaacqWFge=raaa@382D@_2_, ..., G
 MathType@MTEF@5@5@+=feaafiart1ev1aaatCvAUfKttLearuWrP9MDH5MBPbIqV92AaeXatLxBI9gBamrtHrhAL1wy0L2yHvtyaeHbnfgDOvwBHrxAJfwnaebbnrfifHhDYfgasaacH8akY=wiFfYdH8Gipec8Eeeu0xXdbba9frFj0=OqFfea0dXdd9vqai=hGuQ8kuc9pgc9s8qqaq=dirpe0xb9q8qiLsFr0=vr0=vr0dc8meaabaqaciaacaGaaeqabaWaaeGaeaaakeaaimaacqWFge=raaa@382D@_*r *_has to be selected. As these donors are reduced to their specific codon usage tables, we utilized the Codon Usage Database (CUTG) (Release 149.0, September 26, 2005) [35]. Those entries were extracted that consisted of more than 6,400 codons. If a species was represented by more than one table, we took the entry sampling the largest number of codons. This pre-computing phase resulted in the selection of *z *= 690 codon usage tables. Then, a *z *× *z *dissimilarity matrix *D *was set up. For each pair *i*, *j *of species, we calculated the value *D*_*ij *_= 1/2 - *η*_*ij*_. In order to compute the discriminative error *η*_*ij*_, we first considered the set of all "synthetic" genes each comprising 50 codons. Each of the 50 codons was independently selected according to the codon frequencies qc(k)
 MathType@MTEF@5@5@+=feaafiart1ev1aaatCvAUfKttLearuWrP9MDH5MBPbIqV92AaeXatLxBI9gBaebbnrfifHhDYfgasaacH8akY=wiFfYdH8Gipec8Eeeu0xXdbba9frFj0=OqFfea0dXdd9vqai=hGuQ8kuc9pgc9s8qqaq=dirpe0xb9q8qiLsFr0=vr0=vr0dc8meaabaqaciaacaGaaeqabaqabeGadaaakeaacqWGXbqCdaqhaaWcbaGaem4yamgabaGaeiikaGIaem4AaSMaeiykaKcaaaaa@32A4@. We then determined a probability distribution *P*_*k *_for each species *k *on this set. These distributions were utilized to determine *η*_*ij *_in analogy to Equation 4.

Hierarchical divisive clustering [36] was now applied to analyze the dissimilarity matrix *D*. As it was our aim to generate clusters representing taxonomically related species, we used the data basis of the taxonomy browser of the NCBI [37,38] for the following procedure. First, we eliminated all entries, which could not be related to a taxonomical class. Then, we generated for the initiation of the diversification process "class"-clusters consisting of species (i.e. synonymous codon frequency tables) belonging to the same taxonomical class. To test homogeneity of the clusters *G*, we computed for each entry *i *the average dissimilarity dissG(av)
 MathType@MTEF@5@5@+=feaafiart1ev1aaatCvAUfKttLearuWrP9MDH5MBPbIqV92AaeXatLxBI9gBaebbnrfifHhDYfgasaacH8akY=wiFfYdH8Gipec8Eeeu0xXdbba9frFj0=OqFfea0dXdd9vqai=hGuQ8kuc9pgc9s8qqaq=dirpe0xb9q8qiLsFr0=vr0=vr0dc8meaabaqaciaacaGaaeqabaqabeGadaaakeaacqqGKbazcqqGPbqAcqqGZbWCcqqGZbWCdaqhaaWcbaGaem4raCeabaGaeiikaGIaemyyaeMaemODayNaeiykaKcaaaaa@37E4@ (*i*) (see [39]) according to

dissG(av)(i):=1|G\{i}|∑j∈G\{i}Dij.
 MathType@MTEF@5@5@+=feaafiart1ev1aaatCvAUfKttLearuWrP9MDH5MBPbIqV92AaeXatLxBI9gBaebbnrfifHhDYfgasaacH8akY=wiFfYdH8Gipec8Eeeu0xXdbba9frFj0=OqFfea0dXdd9vqai=hGuQ8kuc9pgc9s8qqaq=dirpe0xb9q8qiLsFr0=vr0=vr0dc8meaabaqaciaacaGaaeqabaqabeGadaaakeaacqqGKbazcqqGPbqAcqqGZbWCcqqGZbWCdaqhaaWcbaGaem4raCeabaGaeiikaGIaemyyaeMaemODayNaeiykaKcaaOGaeiikaGIaemyAaKMaeiykaKIaeiOoaOJaeyypa0ZaaSaaaeaacqaIXaqmaeaacqGG8baFcqWGhbWrcqGGCbaxcqGG7bWEcqWGPbqAcqGG9bqFcqGG8baFaaWaaabuaeaacqWGebardaWgaaWcbaGaemyAaKMaemOAaOgabeaakiabc6caUaWcbaGaemOAaOMaeyicI4Saem4raCKaeiixaWLaei4EaSNaemyAaKMaeiyFa0habeqdcqGHris5aaaa@5848@

In order to initiate the split of a cluster *G*, the element *i *∈ *G *having the maximal dissG(av)
 MathType@MTEF@5@5@+=feaafiart1ev1aaatCvAUfKttLearuWrP9MDH5MBPbIqV92AaeXatLxBI9gBaebbnrfifHhDYfgasaacH8akY=wiFfYdH8Gipec8Eeeu0xXdbba9frFj0=OqFfea0dXdd9vqai=hGuQ8kuc9pgc9s8qqaq=dirpe0xb9q8qiLsFr0=vr0=vr0dc8meaabaqaciaacaGaaeqabaqabeGadaaakeaacqqGKbazcqqGPbqAcqqGZbWCcqqGZbWCdaqhaaWcbaGaem4raCeabaGaeiikaGIaemyyaeMaemODayNaeiykaKcaaaaa@37E4@ (*i*) value was chosen. This *i *was the first element of a new cluster *H*. As long as the condition

max⁡k∈G(dissG(av)(k)−dissH(av)(k))≥0
 MathType@MTEF@5@5@+=feaafiart1ev1aaatCvAUfKttLearuWrP9MDH5MBPbIqV92AaeXatLxBI9gBaebbnrfifHhDYfgasaacH8akY=wiFfYdH8Gipec8Eeeu0xXdbba9frFj0=OqFfea0dXdd9vqai=hGuQ8kuc9pgc9s8qqaq=dirpe0xb9q8qiLsFr0=vr0=vr0dc8meaabaqaciaacaGaaeqabaqabeGadaaakeaadaWfqaqaaiGbc2gaTjabcggaHjabcIha4bWcbaGaem4AaSMaeyicI4Saem4raCeabeaakmaabmaabaGaeeizaqMaeeyAaKMaee4CamNaee4Cam3aa0baaSqaaiabdEeahbqaaiabcIcaOiabdggaHjabdAha2jabcMcaPaaakiabcIcaOiabdUgaRjabcMcaPiabgkHiTiabbsgaKjabbMgaPjabbohaZjabbohaZnaaDaaaleaacqWGibasaeaacqGGOaakcqWGHbqycqWG2bGDcqGGPaqkaaGccqGGOaakcqWGRbWAcqGGPaqkaiaawIcacaGLPaaacqGHLjYScqaIWaamaaa@56E1@

was true, the element *k *generating the maximal dissG(av)(k)−dissH(av)(k)
 MathType@MTEF@5@5@+=feaafiart1ev1aaatCvAUfKttLearuWrP9MDH5MBPbIqV92AaeXatLxBI9gBaebbnrfifHhDYfgasaacH8akY=wiFfYdH8Gipec8Eeeu0xXdbba9frFj0=OqFfea0dXdd9vqai=hGuQ8kuc9pgc9s8qqaq=dirpe0xb9q8qiLsFr0=vr0=vr0dc8meaabaqaciaacaGaaeqabaqabeGadaaakeaacqqGKbazcqqGPbqAcqqGZbWCcqqGZbWCdaqhaaWcbaGaem4raCeabaGaeiikaGIaemyyaeMaemODayNaeiykaKcaaOGaeiikaGIaem4AaSMaeiykaKIaeyOeI0IaeeizaqMaeeyAaKMaee4CamNaee4Cam3aa0baaSqaaiabdIeaibqaaiabcIcaOiabdggaHjabdAha2jabcMcaPaaakiabcIcaOiabdUgaRjabcMcaPaaa@4A41@ value was transferred from *G *to *H*. Starting with the initial set of class-clusters described above, the split procedure was applied to that cluster *G *having maximal diameter

diam G:=max⁡i,j∈GDij
 MathType@MTEF@5@5@+=feaafiart1ev1aaatCvAUfKttLearuWrP9MDH5MBPbIqV92AaeXatLxBI9gBaebbnrfifHhDYfgasaacH8akY=wiFfYdH8Gipec8Eeeu0xXdbba9frFj0=OqFfea0dXdd9vqai=hGuQ8kuc9pgc9s8qqaq=dirpe0xb9q8qiLsFr0=vr0=vr0dc8meaabaqaciaacaGaaeqabaqabeGadaaakeaacqqGKbazcqqGPbqAcqqGHbqycqqGTbqBcaaMc8Uaem4raCKaeiOoaOJaeyypa0ZaaCbeaeaacyGGTbqBcqGGHbqycqGG4baEaSqaaiabdMgaPjabcYcaSiabdQgaQjabgIGiolabdEeahbqabaGccqWGebardaWgaaWcbaGaemyAaKMaemOAaOgabeaaaaa@4533@

as long as that maximal diameter was greater than or equal to a threshold *d*_1 _(see [40]).

The procedure resulted in r˜
 MathType@MTEF@5@5@+=feaafiart1ev1aaatCvAUfKttLearuWrP9MDH5MBPbIqV92AaeXatLxBI9gBaebbnrfifHhDYfgasaacH8akY=wiFfYdH8Gipec8Eeeu0xXdbba9frFj0=OqFfea0dXdd9vqai=hGuQ8kuc9pgc9s8qqaq=dirpe0xb9q8qiLsFr0=vr0=vr0dc8meaabaqaciaacaGaaeqabaqabeGadaaakeaacuWGYbGCgaacaaaa@2E28@ = 99 clusters. In order to select a typical example for each cluster, the frequency table having the lowest dissimilarity value to the barycenter of the cluster was chosen. The resulting r˜
 MathType@MTEF@5@5@+=feaafiart1ev1aaatCvAUfKttLearuWrP9MDH5MBPbIqV92AaeXatLxBI9gBaebbnrfifHhDYfgasaacH8akY=wiFfYdH8Gipec8Eeeu0xXdbba9frFj0=OqFfea0dXdd9vqai=hGuQ8kuc9pgc9s8qqaq=dirpe0xb9q8qiLsFr0=vr0=vr0dc8meaabaqaciaacaGaaeqabaqabeGadaaakeaacuWGYbGCgaacaaaa@2E28@ codon usage tables were regarded as representatives for putative sources of aliens genes.

To prevent false predictions, clusters with a composition too similar to the input genome G
 MathType@MTEF@5@5@+=feaafiart1ev1aaatCvAUfKttLearuWrP9MDH5MBPbIqV92AaeXatLxBI9gBamrtHrhAL1wy0L2yHvtyaeHbnfgDOvwBHrxAJfwnaebbnrfifHhDYfgasaacH8akY=wiFfYdH8Gipec8Eeeu0xXdbba9frFj0=OqFfea0dXdd9vqai=hGuQ8kuc9pgc9s8qqaq=dirpe0xb9q8qiLsFr0=vr0=vr0dc8meaabaqaciaacaGaaeqabaWaaeGaeaaakeaaimaacqWFge=raaa@382D@_0 _have to be eliminated. Therefore, the set of r˜
 MathType@MTEF@5@5@+=feaafiart1ev1aaatCvAUfKttLearuWrP9MDH5MBPbIqV92AaeXatLxBI9gBaebbnrfifHhDYfgasaacH8akY=wiFfYdH8Gipec8Eeeu0xXdbba9frFj0=OqFfea0dXdd9vqai=hGuQ8kuc9pgc9s8qqaq=dirpe0xb9q8qiLsFr0=vr0=vr0dc8meaabaqaciaacaGaaeqabaqabeGadaaakeaacuWGYbGCgaacaaaa@2E28@ codon usage tables was preprocessed during the initialization phase for G
 MathType@MTEF@5@5@+=feaafiart1ev1aaatCvAUfKttLearuWrP9MDH5MBPbIqV92AaeXatLxBI9gBamrtHrhAL1wy0L2yHvtyaeHbnfgDOvwBHrxAJfwnaebbnrfifHhDYfgasaacH8akY=wiFfYdH8Gipec8Eeeu0xXdbba9frFj0=OqFfea0dXdd9vqai=hGuQ8kuc9pgc9s8qqaq=dirpe0xb9q8qiLsFr0=vr0=vr0dc8meaabaqaciaacaGaaeqabaWaaeGaeaaakeaaimaacqWFge=raaa@382D@_0_. Those elements were deleted, whose dissimilarity to the frequency table of G
 MathType@MTEF@5@5@+=feaafiart1ev1aaatCvAUfKttLearuWrP9MDH5MBPbIqV92AaeXatLxBI9gBamrtHrhAL1wy0L2yHvtyaeHbnfgDOvwBHrxAJfwnaebbnrfifHhDYfgasaacH8akY=wiFfYdH8Gipec8Eeeu0xXdbba9frFj0=OqFfea0dXdd9vqai=hGuQ8kuc9pgc9s8qqaq=dirpe0xb9q8qiLsFr0=vr0=vr0dc8meaabaqaciaacaGaaeqabaWaaeGaeaaakeaaimaacqWFge=raaa@382D@_0 _was less than a threshold *d*_2_. This procedure resulted in a G
 MathType@MTEF@5@5@+=feaafiart1ev1aaatCvAUfKttLearuWrP9MDH5MBPbIqV92AaeXatLxBI9gBamrtHrhAL1wy0L2yHvtyaeHbnfgDOvwBHrxAJfwnaebbnrfifHhDYfgasaacH8akY=wiFfYdH8Gipec8Eeeu0xXdbba9frFj0=OqFfea0dXdd9vqai=hGuQ8kuc9pgc9s8qqaq=dirpe0xb9q8qiLsFr0=vr0=vr0dc8meaabaqaciaacaGaaeqabaWaaeGaeaaakeaaimaacqWFge=raaa@382D@_0_-specific set of *r *putative sources.

### Testing performance and analyzing genomes

To assess accuracy, SIGI-HMM's predictions were compared with results published in [41]. In nearly all cases, the fraction of pA genes determined by SIGI-HMM was lower; compare results listed in Table 1. This might be due to the focusing of SIGI-HMM on the prediction of GIs. However, for the genome of *Borrelia burgdorferi *SIGI-HMM predicts a significantly higher fraction of pA genes. The organization of this genome is unusual, it consists of 20 mainly linear replicons and is subject to frequent genomic rearrangements [42]. During these reorganization events integration of alien DNA might take place making a larger fractions of pA genes for the *B. burgdorferi *genome plausible. In the following, we report in more detail findings deduced for genomic data sets of the following microbial genomes: *Vibrio cholerae*, *Bacillus subtilis*, *Escherichia coli *K-12, *Methanosarcina mazei*, *Thermus thermophilus *and *Propionibacterium acnes*. The genome of *V. cholerae *consists of two chromosomes with a pronounced asymmetry in the distribution of coding elements with respect to the replicons [43]. Most genes required for growth and virulence are located on chromosome I, whereas chromosome II contains a larger fraction of hypothetical genes.

Interestingly, SIGI-HMM predicted 4.6% pA genes for chromosome I and 21.1% pA genes for chromosome II. Two predicted genomic islands on chromosome I comprise a gene cluster for a toxin-coregulated pilus (VC0813 – VC0845) and fragments of a temperate filamentous phage (VC1455 – VC1457, VC1464, VC1477 – VC1481). Both clusters are closely associated with the pathogenicity of *V. cholerae *[44]. Many of the hypothetical genes encoded on chromosome II are located within a large integron island comprising gene products that might be involved in drug resistance, DNA metabolism and virulence [43]. One of the predicted GIs on chromosome II, which consists of genes VCA0283 – VCA0507, overlaps to a great extent the integron described above. SIGI-HMM identified two additional GIs comprising genes VCA0198 – VCA0202 and VCA0790 – VCA0797, which contain homologs for putative transposases. As transposases are often encoded in genetically mobile IS-elements, these genes are likely candidates for alien genes. For both chromosomes, SIGI-HMM predicts similar distributions of putative donors. The largest fractions belong to the class of bacilli (51% or 61%), whereas the taxonomical class of *V. cholerae*, the *γ*-proteobacteria, accounts for 34% or 37% of all pA genes.

For *B. subtilis*, 10 integrated prophages have been reported (see [4,45,46], and [47]), whose identification is based either on experimental evidence or theoretical considerations. A profound analysis of chromosomal heterogeneities has been accomplished by Nicolas *et al. *[9], using a HMM on the nucleotide level. All genomic islands identified by Nicolas *et al*. were largely confirmed by SIGI-HMM. Both approaches detected nine of the putative prophages and several other islands assigned to functions in cell wall biosynthesis, competence and resistance. In contrast to Nicolas *et al*., SIGI-HMM identified pA genes, which belong to the experimentally reported integrated prophage PBSX [47]. In summary, SIGI-HMM predicted for *B. subtilis *9.5% of the genes as being pA, most of them originating from the class of bacilli (316 pA genes, 81%).

Based on a combination of parameters measuring computational complexity, Lawrence and Ochman [4] had estimated that about 18% of the *E. coli *K-12 genome have been imported *via *lateral gene transfer. In contrast, SIGI-HMM predicted 580 (13.4%) pA genes which were mostly organized in small clusters of less than ten genes. 521 pA genes (92%) seem to originate from *γ*-proteobacteria, the taxonomical class *E. coli *belongs to. The largest GIs included the cryptic prophages CP4-6 (262 – 297 kbp), DLP12 (557 – 584 kbp), e14 (1,196 – 1,221), Rac (1,410 – 1,433 kbp), Qin (1,631 – 1,651 kbp), CP4-44 (2,064 – 2,069 kbp), CPS-53 (2,465 – 2,475 kbp), Eut (2,556 – 2,563 kbp), CP4-57 (2,752 – 2,775 kbp), and the phage-like element KpLE2 (4,494 – 4,544 kbp) (for review see [48]). 44 IS-elements have been annotated within the genome of *E. coli *K-12, SIGI-HMM predicted 34 of them correctly.

*T. thermophilus *is an extreme thermophilic bacterium living as a halotolerant in an extreme ecological niche. Two *T. thermophilus *strains, namely HB27 [45] and HB8 [46], have been sequenced so far. SIGI-HMM predicted for both strains a small fraction of pA genes (HB27 1.0%; HB8 1.7%). The largest pA cluster consists of 6 genes in case of HB27 (TTC0277 – TTC0278, TTC0280 – TTC0283) and of 5 genes in case of HB8 (TTHA0644 – TTHA0648). The GIs share no sequence similarity and contain genes that are associated with functions in cell wall biosynthesis. Most pA genes seem to originate from the class of the *δ*-proteobacteria (HB27 5 genes; HB8 18 genes). In both genomes no donor was predicted for 12 pA genes, respectively.

It has been suggested that HGT plays an important role in the evolution of the mesophilic archaeon *M. mazei *[49]. The analysis of protein sequences *via *BLAST showed that 31% of the archeal sequences were more similar to bacterial than to archeal ones. SIGI-HMM predicted for *M. mazei *only 8.4% pA genes. Please note that the two analyses used different approaches for pA prediction and that SIGI-HMM focuses on the analysis of GIs only. These systematic differences may explain the findings. Interestingly and in agreement with the above analysis, only 21% of the pA genes seem to originate from the archeal domain. 27% of the pA genes were predicted to originate from the class of shingobacteria, 23% from chlamydia and 11% from clostridia. This finding is also in agreement with the postulated gene flux from mesophilic bacteria to mesophilic archaea [27].

*P. acnes *is a major inhabitant of the adult human skin, living in sebaceous follicles [50]. Usually the bacterium is harmless; however it is involved in acne vulgaris formation. The genome harbors genes whose products are involved in degrading host molecules and pore-forming factors. It also contains surface-associated and other immunogenic factors, which might be responsible for acne inanimation and other *P. acnes*-associated diseases. SIGI-HMM predicted 4.1% pA genes clustered in five larger GIs and several smaller islands of less than five genes. 47% (45 genes) of them are predicted to originate from the *α*-proteobacteria, but only 13% (12 pA genes) from the taxonomic class of *P. acnes*, the actinobacteria. Interestingly, four of the larger GIs and two of the smaller islands are flanked by tRNA-genes in direct or close vicinity. tRNAs are considered to be hot spots for recombination events that can result in horizontal gene transfer. SIGI-HMM found these anomalies although it does not interpret sequences besides protein coding genes. Of the larger GIs, the first (at position 28 – 34 kbp) contains genes without functional assignment, the second (874 – 880 kbp) harbors genes for several transport systems among others for iron(III)dicitrate (PPA0792 – PPA0794) and the third (921 – 941 kbp) for an ABC-type transport system (PPA0843 – PPA0845), putative conjugal transfer proteins (PPA0846 – PPA0848) and two putative transposases (PPA2354, PPA0858). The forth GI (1,390 – 1,407 kbp) contains a gene cluster for a putative non-ribosomal peptide synthetase (NRPS) (PPA1287 – PPA1290). NRPSs are involved in the biosynthesis of complex secondary metabolites. As many of the genes clustered in the fifth GI (1,707 – 1,731 kbp) are annotated as phage-associated proteins (PPA1593 – PPA1596, PPA1604 – PPA1605), the GI may be attributed to an integrated prophage.

**Figure 2:**
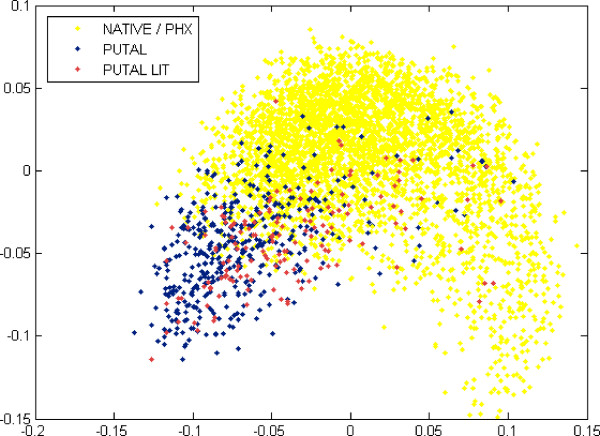
Kernel-based scatter plot visualization of SIGI-HMM predictions for *E. coli *K-12. Blue points (PUTAL) represent pA genes as predicted by SIGI-HMM, red points (PUTAL LIT) indicate predicted pA genes with additional evidence from the current literature as described in the text. Yellow points (NATIVE / PHX) refer to genes which are predicted to be native or highly expressed.

**Figure 3:**
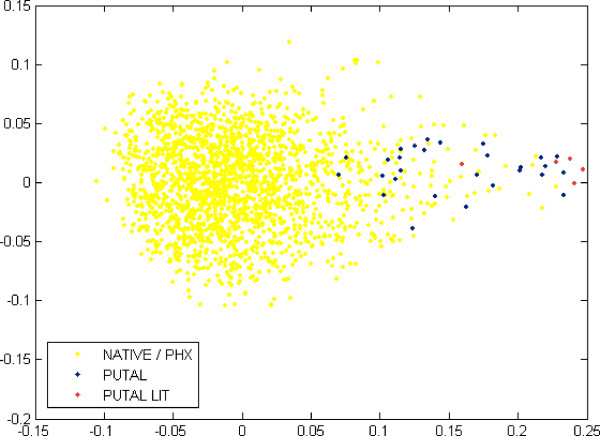
Kernel-based scatter plot visualization of SIGI-HMM predictions for *T. thermophilus*. Blue points (PUTAL) represent pA genes as predicted by SIGI-HMM, red points (PUTAL LIT) indicate predicted pA genes with additional evidence from the current literature as described in the text. Yellow points (NATIVE / PHX) refer to genes which are predicted to be native or highly expressed.

**Figure 4:**
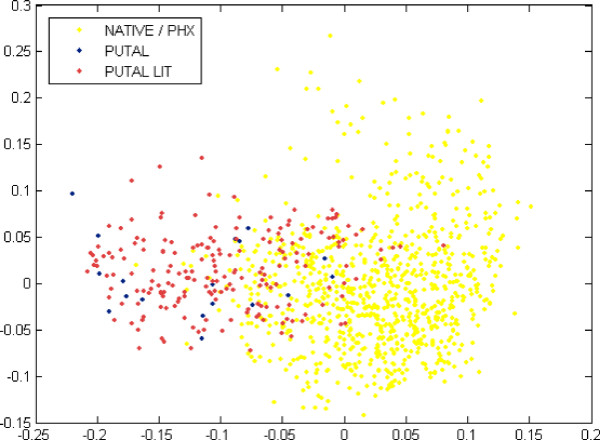
Kernel-based scatter plot visualization of SIGI-HMM predictions for *V. cholerae *(chromosome II). Blue points (PUTAL) represent pA genes as predicted by SIGI-HMM, red points (PUTAL LIT) indicate predicted pA genes with additional evidence from the current literature as described in the text. Yellow points (NATIVE / PHX) refer to genes which are predicted to be native or highly expressed.

For visualization of the HMM-based predictions we use scatter plot repesentations providing an overview of codon usage similarities between all genes of a genome. By means of a newly developed kernel for measuring similarity of codon usage tables [51], we perform a kernel principal component analysis (see e.g. [52]) to compute the resulting 2D coordinates of all genes. In that representation, nearby points indicate a similar codon usage of the corresponding genes. It is important to note that the kernel-based approach does not use any information about the location of genes on the genome. Instead, codon usage correlations between different amino acids are used to derive the two-dimensional representation. This approach is different from the concept of SIGI-HMM. Therefore, a clustering of SIGI-HMM predicted pA genes which becomes visible in the scatter plots (see Figure 2, 3, and 4) confirms the corresponding predictions.

Figure 2 is a plot of all genes of the *E. coli *K-12 genome. The general form resembles the "rabbit head" trimodal shape described earlier for the genome of *B. subtilis *[53]. Most genes belonging to integrated prophages are located in the lower left "ear". PHX genes are clustered in the lower right corner.

*T. thermophilus *is one of the genomes with lowest pA content. The plot depicted in Figure 3 represents the genome of *T. thermophilus *and has a quite specific shape. This finding indicates that the overall shape of the plot is massively modulated by the fraction of genes acquired *via *HGT. The pA genes as predicted by SIGI-HMM are mainly located in a long tail with low point density on the right hand side of the plot.

As already mentioned, the genome of *V. cholerae *consists of two chromosomes. Most essential genes are located on chromosome I and codon usage of genes on chromosome II is rather inhomogeneous. Again, the overall shape of the plot, which represents chromosome II, reflects this situation (compare Figure 4) and shows a well-clustering fraction of pA genes located in the lower left corner of the plot. Please note that the positioning of pA genes predicted by SIGI-HMM only and those pA genes supported by additional evidence from the literature corresponds to a great extent in all plots.

### Assessing the patchiness of GIs

Genomic islands are thought to be the result of constant genetic rearrangement events, which account for their observed mosaic structure. As these rearrangements could also take place at hot spots for the integration of alien DNA in the host genome, patches of genes having a codon usage similar to the host have to be expected inside GIs. This fact makes it difficult to determine the number of false negatives, even in annotated GIs. The number of false positives is difficult to deduce too, as it is hard to proof that a stretch of pA genes has not been acquired *via *HGT. In order to illustrate the problem and the patchiness of GIs, we compare in more detail some predictions with published findings.

Chromosome II of *V. cholerae *contains an integron island of size 125.3 kbp, which includes genes VCA0271 to VCA0491 [43]. Of these 214 genes, SIGI-HMM labels 188 as pA (87%), 1 as AT (atypical) and 25 as pN (putatively native). SIGI-HMM did subdivide the integron island into the following patches: VCA0271 – VCA0282 pN, VCA0283 – VCA0286 pA, VCA0287 – VCA0291 pN, VCA0292 – VCA0324 pA, VCA0325 – VCA0329 pN, VCA0330 – VCA0379 pA, VCA0380 – VCA0385 pN, VCA0386 – VCA0507 pA. From the remaining 611 genes on the chromosome, 42 were predicted as pA.

The chromosome of *Mesorhizobium loti *consists of 6.725 protein coding genes. It contains a 611 kbp DNA segment which is, as the authors put it, "a highly probable candidate of a symbiotic island" [3]. SIGI-HMM predicted 5.561 genes as pN, 1.161 (17%) as pA and 30 as AT. Of the symbiotic island, 145 genes were pN, 421 pA (72%) and 14 AT. The pA genes were clustered in 29 GIs ranging in size from 2 to 108 genes.

As already mentioned, ten integrated prophages or prophage-like elements were reported for the genome of *B. subtilis *[9]. Five of these elements are flanked by sequence repeats which we considered as the original integrations sites indicating the actual borders of the GIs. Table 2 summarizes composition and location of related GIs predicted by SIGI-HMM. Skin prophage and P7 have a mosaic structure and harbour ≈ 50% pN genes. In four of the five cases, the borders of the predicted GIs are in good agreement with the location of the repeats.

## Discussion

### Analysis of codon usage reliably allows to identify most HGT events

We have to stress that our approach entirely relies on the analysis of codon usage. SIGI-HMM does not interpret additional signals like direct repeats or disrupted tRNA sequences frequently flanking GIs. Therefore, the outcome of the HMM analysis are DNA regions showing atypical codon usage. This fact has two consequences: 1) SIGI-HMM is unable to identify GIs having an unsuspicious codon usage and 2) the rationale of naming these stretches GIs merely depends on the correlation with biological findings.

However, we have shown that DNA regions identified by SIGI-HMM as suspicious correspond to known cases of horizontally transferred elements like phages. Our approach of focusing on the analysis of codon usage is not a completely new one. There exist several methods to identify horizontally transferred genes. These approaches rely on the analysis of codon or amino acid sequences or the construction of phylogenetic trees. For a comparison see e.g. [14]. Each approach has individual drawbacks and it might be that each method identifies a specific class of genes acquired in a different time of genome evolution [13]. It was argued that codon usage is no reliable indicator for the study of HGT [54]. However, it was shown that related methods identify pA genes to a great extent [55]. The assumption that methods analyzing codon usage might overlook horizontally acquired genes could be valid for more ancient events. For these genes, the effect of amelioration [56] might have rendered codon usage unsuspicious. Lawrence and Ochman estimated the age of imported genes [4]. Their conclusion was that most were relatively recent, i.e. acquired within the last few million years; see also [57]. This suggests that older imports have been purged presumably because the acquired genes did not improve fitness. If this argument is true, there is no need to search for larger amounts of ancient pA genes. Therefore, methods based on the analysis of codon usage should have the potential of identifying a great fraction of horizontally transferred genes. Low values of pA content can frequently be explained with biological findings. It was argued that species populating extreme ecological niches tend to have relative small genomes [58]. The size of the sequenced *T. thermophilus *genomes support this notion. If selective pressure minimizes genome size, it will also effect acquisition and conservation of foreign DNA. The low fraction of pA genes determined for both strains is in agreement with the above hypothesis.

The methods will fail at alien genes having a codon usage undistinguishable from the host's preferences. Among them might be ancient pA genes. Because of the amelioration process, ancient pA genes are harder to detect. These pA genes, surviving the selection process may actually constitute important and useful genes. In order to complete the set of identified HGT events and to reduce the number of false negatives, it will be necessary to use a completely different approach like the construction of phylogenetic trees.

If not processed correctly, highly expressed genes could be a source for false positive predictions. It is known that these genes show a distinct codon usage by preferring a species-specific set of major codons. In order to reduce the rate of false positive predictions, we use a filter which is based on a method [30] shown to be effective in predicting gene expressivity [31]. We have adjusted the parameters (see Equation 4) in such a way that the errors of the first and second kind are equally likely. Highly expressed genes belong to the core of a genome and it is unlikely that these genes are subject to HGT. Nevertheless, the user may disable this filter in order to study its influence on GI prediction.

### Focusing on the prediction of GIs is biologically reasonable and reduces the risk of false predictions

Intrinsically, increasing the sensitivity of a test also increases the risk of predicting false positives. For the prediction of pA genes, the risk can however be minimized, if an algorithm focuses on the prediction of genomic islands as SIGI-HMM does. The pieces of DNA acquired *via *HGT typically have a considerable length. Examples are the symbiotic island of size 611 kbp described for the genome of *M. loti *or the integron island of size 125 kbp found on chromosome II of *V. cholerae *(see Results). Genes responsible for pathogenicity are also agglomerated in islands; see [2] and references therein. Therefore, a focusing on predicting GIs rather than all pA genes is an appropriate strategy to avoid false positives without missing relevant HGT events. Consequently, this argument was considered for the design of recently introduced algorithms [23,59]. However, the rate of false positive predictions will increase, if codon usage of a genome is inhomogeneous. To avoid this situation, it is important, to determine the CSC of a genome.

### Codon usage is a reliable indicator to predict the origin of pA genes

For each completely sequenced genome, we have computed a variant of the CSC defined above; see [60]. It consisted of those genes having a homogeneous codon usage. The results obtained with the classification of genes from CSCs show that codon usage hints at the origin of genes. First tests indicate that prediction quality is high, as long as the CSC contains at least 70% of the genes. In addition, the results of performance tests (see [23]) carried out to demonstrate SIGI's ability of predicting the putative donor are also valid for SIGI-HMM.

Omelchenko *et al*. [61] used BLAST on the protein level to determine HGT events in the genome of *T. thermophilus *HB27. The protein sequences of many genes were similar to those of hyperthermophilic archaea. Taxonomical classification of donors for genes constituting GIs predicted by SIGI-HMM was rather inhomogeneous. The putative donors belonged to bacteria, archaea and eukaryota. It will be necessary to evaluate methods for pA prediction with a standardized test bed. Artificial genomes as introduced recently [62] may constitute the basis for such a validation, which may lead to a contest of methods for pA prediction.

## Conclusion

An inhomogeneous HMM on gene level allows to identify GIs in microbial genomes and to predict the putative donor of horizontally transferred genes. The predictions are consistent with known findings and do not depend on the optimization of many parameters. Our implementation as a freely available tool written in Java allows an independent inspection of genomes in great detail. The genome-specific predictions can be used for further analysis or the comparison of several methods.

## Authors' contributions

SW and RM specified the problem and the solution strategy. SW developed the HMM together with OK and KS and provided resources. OK and TB implemented the HMM. The donor selection was conceptualized by SW, RA and CD, and implemented by RA. WFF analyzed the genomes. PM decisively contributed to the methods measuring similarity of codon usage tables. RM conducted the performance tests and contributed substantially to the manuscript which was prepared together with SW and KS. All authors read and approved the final manuscript.

## Figures and Tables

**Table 1:** A comparison of pA predictions for prokaryotic species. SIGI-HMM was used to identify GIs. The accumulated length of genes constituting GIs is given in percent in column pA DNA. This transformation allows to compare results with entries of column Foreign DNA, which was reproduced from [41]. The column Length lists the genome size im Mbp.

Species	Length [Mbp]	Foreign DNA [%]	pA DNA [%]
*Escherichia coli *K-12	4.64	12.8	9.3
*Bacillus subtilis*	4.21	7.5	7.6
*Synechocystis *PCC6803	3.57	16.6	5.0
*Deinococcus radiodurans*	2.65	5.2	4.8
*Archaeoglobus fulgidus*	2.18	5.2	4.2
*Aeropyrum pernix*	1.67	3.2	1.5
*Thermotoga maritima*	1.86	6.4	1.0
*Pyrococcus horikoshii*	1.74	2.7	2.9
*Methanobacterium thermoautotrophicum*	1.75	9.4	1.6
*Haemophilus influenzas *Rd KW20	1.83	4.5	1.6
*Helicobacter pylori *26695	1.67	6.2	0.0
*Aquifex aeolicus*	1.55	9.6	1.8
*Methanocaldococcus jannaschii*	1.66	1.3	0.2
*Treponema pallidum*	1.14	3.6	0.3
*Borrelia burgdorferi*	0.91	0.1	8.5
*Rickettsia prowazekii*	1.11	0.0	0.0
*Mycoplasma pneumoniae*	0.82	11.6	3.8
*Mycoplasma genitalium*	0.58	0.0	0.2

**Table 2:** Prophages and prophage-like elements integrated into the genome of *B. subtilis*. Column 1 lists the elements flanked by sequence repeats. Column 2 gives the location of the repeats. Column 3 and 4 list the number of pA and pN genes predicted for these GIs by SIGI-HMM. The two last columns indicate the offset of the GI from the sequence repeats. An offset of -1 means that the GI predicted by SIGI-HMM starts (ends) one gene after (before) the repeat. Positions are as in [9] and given in kbp.

Element	Repeats	# pA	# pN	Offset Begin	Offset End
P1	202 – 213	10	1	0	-1
P2	555 – 567	10	1	-1	0
P6	2050 – 2060	9	0	0	0
Skin	2654 – 2701	32	31	0	0
P7	2725 – 2735	7	6	-6	0
